# Synthesis and Antimycobacterial Activity of 3-Phenyl-1*H*-indoles

**DOI:** 10.3390/molecules26175148

**Published:** 2021-08-25

**Authors:** Renata Jardim Etchart, Raoní S. Rambo, Bruno Lopes Abbadi, Nathalia Sperotto, Christiano Ev Neves, Fernanda Fries Silva, Maiele Dornelles, Lovaine Duarte, Fernanda Souza Macchi, Marcia Alberton Perelló, Rogério Vescia Lourega, Cristiano Valim Bizarro, Luiz Augusto Basso, Pablo Machado

**Affiliations:** 1 Centro de Pesquisas em Biologia Molecular e Funcional, Instituto Nacional de Ciência e Tecnologia em Tuberculose, Pontifícia Universidade Católica do Rio Grande do Sul, Avenida Ipiranga, 6681-Prédio 92A, Porto Alegre 90616-900, RS, Brazil; renata.etchart@gmail.com (R.J.E.); raoni.rambo@gmail.com (R.S.R.); br.abbadi@gmail.com (B.L.A.); nathalia.sperotto@gmail.com (N.S.); christiano_neves@hotmail.com (C.E.N.); fernanda.fries@acad.pucrs.br (F.F.S.); maieledornelles@gmail.com (M.D.); lovaduarte@gmail.com (L.D.); fernanda.macchi@outlook.com (F.S.M.); marcia.perello@pucrs.br (M.A.P.); cristiano.bizarro@pucrs.br (C.V.B.); luiz.basso@pucrs.br (L.A.B.); 2 Programa de Pós-Graduação em Biologia Celular e Molecular, Pontifícia Universidade Católica do Rio Grande do Sul, Porto Alegre 90616-900, RS, Brazil; 3 Instituto de Química, Departamento de Química, Universidade Federal do Rio Grande do Sul, Porto Alegre 91501-970, RS, Brazil; louregarv@gmail.com; 4 Programa de Pós-Graduação em Medicina e Ciências da Saúde, Pontifícia Universidade Católica do Rio Grande do Sul, Porto Alegre 90616-900, RS, Brazil

**Keywords:** 1*H*-indoles, *Mycobacterium tuberculosis*, mammalian cellular viability, genotoxicity, time-kill, pharmacodynamic model

## Abstract

Tuberculosis has been described as a global health crisis since the 1990s, with an estimated 1.4 million deaths in the last year. Herein, a series of 20 1*H*-indoles were synthesized and evaluated as in vitro inhibitors of *Mycobacterium tuberculosis* (Mtb) growth. Furthermore, the top hit compounds were active against multidrug-resistant strains, without cross-resistance with first-line drugs. Exposing HepG2 and Vero cells to the molecules for 72 h showed that one of the evaluated structures was devoid of apparent toxicity. In addition, this 3-phenyl-1*H*-indole showed no genotoxicity signals. Finally, time-kill and pharmacodynamic model analyses demonstrated that this compound has bactericidal activity at concentrations close to the Minimum Inhibitory Concentration, coupled with a strong time-dependent behavior. To the best of our knowledge, this study describes the activity of 3-phenyl-1*H*-indole against Mtb for the first time.

## 1. Introduction

Tuberculosis (TB) is an ancient disease, dating back to 70,000 years ago, and remains a global public health emergency causing more than 1 million deaths each year. According to the WHO Global Tuberculosis Report (2020), an estimated 10.0 million people fell ill with TB in 2019, with 1.2 million deaths among HIV-negative people and an additional 208,000 deaths among HIV-positive people [1].

After initial exposure, the intracellular pathogen *Mycobacterium tuberculosis* (Mtb), the causative agent of human TB, is absorbed by the alveolar macrophages of the lung. At this point, most infections are eliminated by a functioning immune system. If there is uncontrolled bacterial replication, a primary infection is established. This can develop into an active infection, which can lead to the development of symptoms of the disease (cough, night sweats, weight loss, and so forth) [2].

The first-line treatment of TB consists of a regimen of four drugs (isoniazid, rifampicin, ethambutol, and pyrazinamide), lasting 6 months. TB is a particularly challenging disease, with rapid development of resistant strains, mainly attributed to the low adherence to treatment protocols. Multidrug-resistant TB (MDR-TB) is caused by bacilli that are resistant to at least isoniazid and rifampin, whereas extensively drug-resistant TB (XDR-TB) is occasioned by resistant strains to isoniazid and rifampin, plus any fluoroquinolone and at least one of three injectable second-line drugs [3,4].

The discovery of drugs such as isoniazid and pyrazinamide (discovered in the early 1950s), ethambutol and rifampicin (discovered in the 1960s) remains the basis of anti-TB therapy. After 1960, there was no completely new drug to treat TB until bedaquiline was approved in 2012 [5,6]. A few years later, the drugs delamanid (2014) and pretomanid (2019) were also approved. These three approved drugs received indications for the treatment of MDR-TB [2,4].

Despite the capacity of these new pharmacological alternatives to treat TB, the high capability for adaptation and the development of mutations associated with resistance [7] have highlighted the importance of continuous efforts to discover and develop new anti-TB drugs. Our research group has synthesized and evaluated different heterocyclic compounds against drug-susceptible and drug-resistant Mtb strains, with some encouraging results [8,9,10]. Importantly, the indole scaffold has been described in compounds endowed with antitubercular activity [11]. Additionally, this heterocycle has been obtained in molecules with antiviral, anti-inflammatory, antinociceptive, anticancer, anti-HIV, antioxidant, antimicrobial, antidiabetic, antimalarial, and anticholinesterase activities [12]. However, to the best of our knowledge, 3-phenyl-1*H*-indoles have not yet been evaluated as possible inhibitors of Mtb growth.

Within this context, the present study describes the synthesis of 3-phenyl-1*H*-indoles and their in vitro activity against drug-susceptible and drug-resistant Mtb strains. Additionally, apparent cytoxicity and genotoxicity were studied using mammalian cells. Finally, antimycobacterial kinetic behavior was also shown, with concomitant exploration of a pharmacodynamic model.

## 2. Results

Compounds were synthesized by a well-established protocol for direct arylation of *NH*-indoles [13]. The method was highly regioselective, allowing 3-phenyl-1*H*-indoles to be obtained in a catalytic system based on palladium(II) acetate/bis(diphenylphosphino)methane [Pd(OAc)_2_/dppm] using water as solvent. The palladium-catalyzed procedure led to C-H functionalization of indoles **1**, with aryl halides **2** leading to the desired compounds **3a**–**t** with 10–77% yields after purification (Scheme 1). The spectroscopic and spectrometric data obtained were in full agreement with the proposed structures (Section 3.2 and Supplementary Materials Figures S1–S40).

**Scheme 1:**
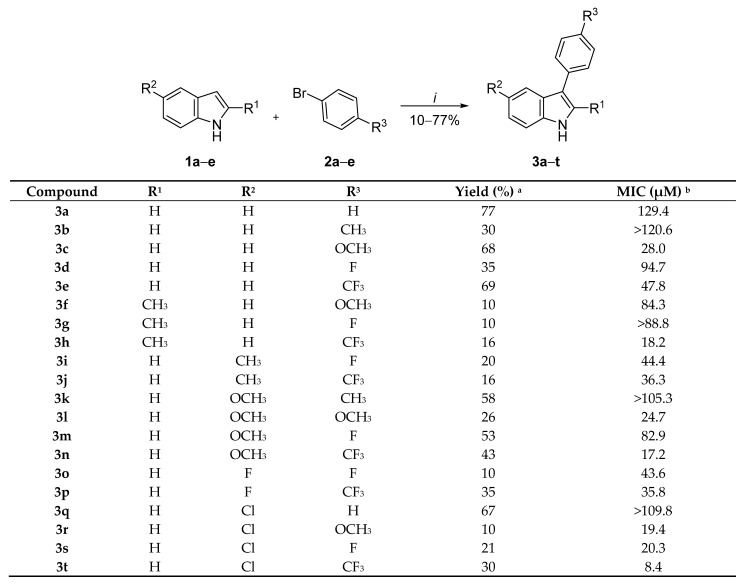
Reagents and conditions: *i* = Pd(OAc)_2_, dppm, LiOH·H_2_O, H_2_O, 110 °C, 24 h. ^a^ Purified products. ^b^ *M. tuberculosis* H37Rv strain. MIC = Minimum Inhibitory Concentration. Data were obtained from three independent experiments and the most frequent MIC value was showed.

The synthesized 3-phenyl-1*H*-indoles **3a**–**t** were evaluated in a whole-cell assay against the Mtb H37Rv strain using isoniazid as positive control [9] (Scheme 1). Under our experimental conditions, this first-line drug presented a Minimum Inhibitory Concentration (MIC) of 2.3 µM. In general, the MIC values exhibited by the compounds seemed to have a low correlation with the electronic and physicochemical characteristics of the substituents. The results indicated that steric components seem to be more important in explaining, even partially, the bacterial growth inhibition potencies.

The unsubstituted molecule **3a** was inactive at the highest concentration evaluated (129.4 µM). The presence of a methyl group at the 4-position of the benzene ring also provided an inactive structure, as the 1*H*-indole **3b** presented a MIC > 120.6 µM. The increase in molecular volume with the concomitant insertion of an atom that can act as a hydrogen bond acceptor increased antimycobacterial activity since the 4-methoxy-containing compound **3c** exhibited a MIC value of 28.0 µM. By contrast, the presence of a substituent with a reduced molecular volume and electron withdrawing character attached at the 4-position of the benzene ring reduced the molecular ability to inhibit Mtb growth. The fluorinated structure **3d** showed a MIC value of 94.7 µM. Interestingly, when the fluorine atom was replaced with a trifluoromethyl group in the 1*H*-indole **3e**, the antimycobacterial activity was almost doubled, reducing the MIC value to 47.8 µM.

In the second round of structural modifications, the presence of a methyl group at the 2-position of the heterocyclic ring was studied. Furthermore, the substituents that produced compounds with higher activity (4-OCH_3_, 4-F, and 4-CF_3_) were kept on the benzene ring. The methoxy-substituted molecule **3f** was able to inhibit the growth of the bacillus with a MIC of 84.3 µM. Once again, the presence of the fluorine atom on the benzene ring in the **3g** structure reduced the activity. By contrast, the trifluoromethyl group attached at the 4-position of the benzene ring of the 1*H*-indole **3h** produced a compound with a MIC of 18.2 µM. Comparing the inhibitory capacity of the molecules in the presence (**3h**) and absence (**3e**) of the methyl group attached at the 2-position of the indole ring, one can conclude that **3h** was approximately 2.6-fold more effective than **3e**.

Finally, attention was turned to the 5-position of the indole ring. The first substituent evaluated was a methyl group at that position. Compounds **3i** and **3j**, containing fluorine and trifluoromethyl group attached at the benzene ring, presented MIC values of 44.4 µM and 36.3 µM, respectively. The methoxy group was also evaluated at the 5-position of the heterocyclic ring in molecules **3k**–**n**. The presence of the methyl group on the benzene ring of the **3k** structure led to an inactive compound, considering the highest evaluated concentration. This was the same pattern observed for the methylated 1*H*-indole **3b**. Furthermore, the dimethoxy-substituted compound **3l** exhibited a MIC value of 24.7 µM. This activity was similar to that of the monosubstituted molecule **3c** (28.0 µM). When the fluorine atom replaced the methoxy group on the benzene ring, the antimycobacterial activity was reduced more than three-fold, as the MIC value of the 1*H*-indole **3m** was 82.9 µM. By contrast, the trifluoromethyl group produced an increased inhibitory activity on Mtb. Compound **3n** showed a MIC of 17.2 µM, which was slightly lower than that shown by the **3l** structure. When the fluorine atom was used as a substitute attached to the 5-position of the indole ring, the resulting molecules did not show significant variations in their activity against the bacillus. The 1*H*-indoles **3o** and **3p** presented MIC values of 43.6 µM and 35.8 µM, respectively. Finally, the chlorine atom was studied attached at the 5-position of the indole ring in compounds **3q**–**t**. As demonstrated above for molecule **3a**, the absence of substituents on the benzene ring significantly reduced antimycobacterial activity. Thus, the 1*H*-indole **3q** was inactive at the highest concentration tested (>109.8 µM). Once again, the presence of the methoxy group produced an effective molecule, which was able to inhibit Mtb growth. The **3r** structure exhibited an MIC of 19.4 µM, a value that ranks it among the most effective in the series examined. The fluorine-containing molecule **3s** showed a similar ability to inhibit the bacillus, with an MIC of 20.3 µM. Last but not least, the presence of the trifluoromethyl group on the benzene ring and the chlorine atom attached at the 5-position of the heterocycle led to the structure with the highest activity in the synthesized series. Compound **3t** was able to inhibit Mtb growth with an MIC value of 8.4 µM. It is important to mention that trifluoromethyl group has unique physicochemical properties, increasing lipophilicity while reducing the reactivity of the compounds in metabolizing reactions. Particularly, greater lipophilicity may be related to the greater antimycobacterial activity of trifluoromethylated-1*H*-indoles (Scheme 1).

The most effective compounds against the H37Rv strain (**3h**, **3n**, **3r**, and **3t**) were evaluated against a well-characterized panel of clinical isolates described as multidrug-resistant Mtb strains (Table 1). The PT2, PT12, and PT20 strains have been described as resistant to drugs such as isoniazid, rifampin, streptomycin, ethionamide, and rifabutine. In addition, PT12 and PT20 are also resistant to drugs such as pyrazinamide and ethambutol, and PT12 exhibits additional resistance to amikacin and capreomycin. Importantly, the genomes of these mycobacterial strains have been fully sequenced; therefore, genetic modifications related to resistance phenotypes are known [14]. Interestingly, the 1*H*-indoles **3h**, **3n**, **3r** and **3t** maintained the MIC value against the drug-resistant Mtb strains or were even more effective, with MIC values lower than those exhibited against the H37Rv strain. These findings demonstrate that this class of molecules does not share cross-resistance with some of the main drugs used for the clinical management of TB. Moreover, the results suggest that this chemical class may provide active compounds against drug-susceptible and drug-resistant Mtb strains, acting by distinct mechanisms of action when compared to first-line drugs.

The viability of the HepG2 and Vero cells in the presence of the compounds was determined in order to evaluate the selectivity and provide preliminary evidence of the toxicity of the molecules. Cell viability was assessed at a mitochondrial (MTT) [15] and lysosomal (Neutral Red) [16] level after exposure to the 1*H*-indoles **3h**, **3n**, **3r** and **3t** for 72 h (Table 1). The results were expressed as the concentration capable of reducing cell viability by 50% (CC_50_). 1*H*-Indoles **3h**, **3n**, and **3t** inhibited the viability of both cell lines at concentrations less than 30 µM. This finding denotes that these molecules have a possible propensity for toxicity and a low selectivity, considering the relationship between mammalian cells (HepG2 and Vero) and mycobacterial (Mtb) cells. By contrast, compound **3r** at 30 µM concentration did not significantly affect the viability of either cell lineage. Based on these results, the 1*H*-indole **3r** was chosen for subsequent trials.

**Figure 1:**
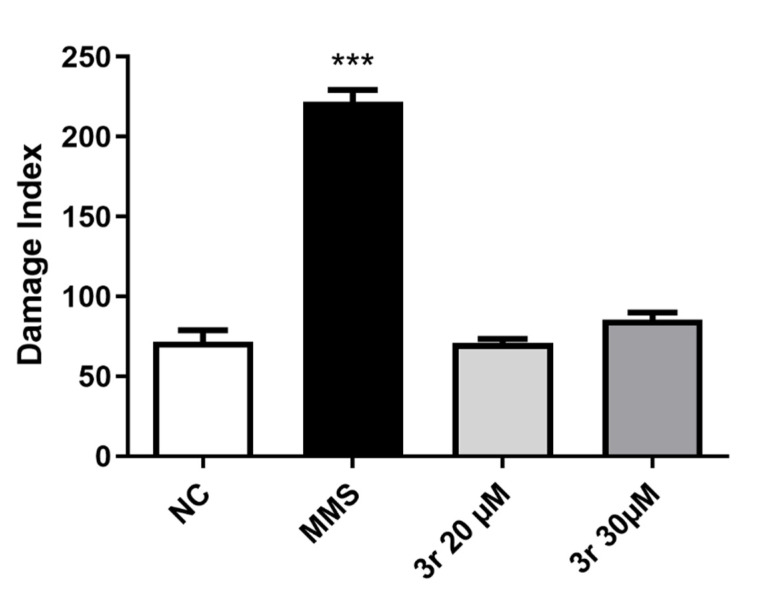
DNA damage index measured by alkaline comet assay in HepG2 cells incubated for 24 h with **3r**. NC = control vehicle group (0.5% DMSO), MMS = methyl methanesulfonate, positive control group. Data are expressed as the mean ± standard error from two independent experiments. Statistical analysis was performed by one-way analysis of variance followed by Dunnett’s post-test. *** *p* < 0.001.

Additionally, the genotoxicity of compound **3r** was studied, as cell viability results could indicate a certain degree of toxicity in the series of molecules evaluated. Therefore, the induction of DNA single and double-strand breaks and alkali-labile sites in HepG2 cells were evaluated using the alkaline comet assay [17]. The results demonstrated that compound **3r** did not produce DNA damage at the evaluated concentrations (Figure 1). These results indicate a reasonable safety profile for 1*H*-indole **3r** based on cytotoxic and genotoxic assessments.

**Figure 2:**
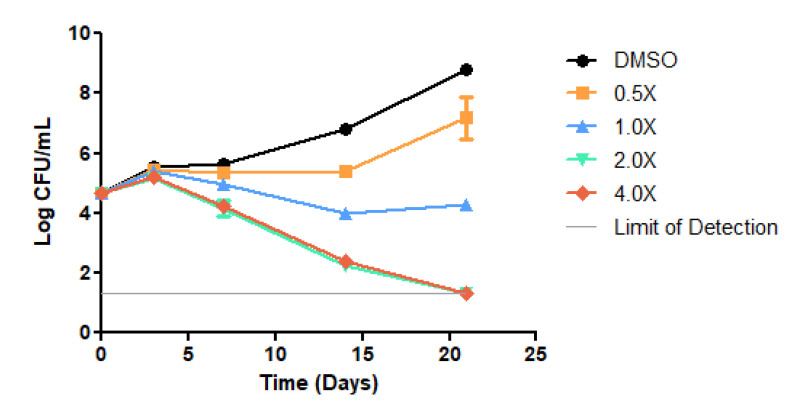
Time-kill curves of compound **3r** against replicating *Mycobacterium tuberculosis* H37Rv. Kill kinetics were determined under standard aerobic conditions. Viability was monitored by determining CFU/mL. The lower limit of detection was 200 CFU/mL for undiluted samples on plates. Data represent the mean ± standard deviation of biological triplicate measurements.

The determination of the MIC of an antimicrobial drug is widely used as a marker of its effect. However, since the MIC value is obtained at a single time point, there is some information about other aspects of the antibiotic’s effect that may be relevant, such as combinations of growth and kill rates, the persistent activity of the compound, and its effect over time. Thus, time-kill analysis of 1*H*-indole **3r** was performed to assess the dynamics of the antimycobacterial activity [18,19,20] (Figure 2). Compound **3r** showed time-dependent kill kinetics at the highest evaluated concentrations. On day zero, the average CFU/mL value was around 5 logs. The vehicle (DMSO) showed growth, with values around 8.8 logs on day 21. Using a concentration of 0.5 × MIC of compound **3r**, a regrowth of the culture was observed on day 21 (around 6.3 logs). However, this value was lower than that presented by the DMSO. 1*H*-Indole **3r** at a concentration of 1.0 × MIC value was able to maintain the culture in the range of 4.6 to 4.2 logs over the 21 days. Taken together, these results denote that concentrations of 0.5 × MIC (10 µM) and 1.0 × MIC (20 µM) of **3r** exhibited bacteriostatic effects over 21 days. In addition, the Minimum Bactericidal Concentration (MBC) was 2 × MIC (40 µM), which reduced the culture count to around 2.3 logs on day 14 and below the detection limit on day 21. It is noteworthy that there was no antimycobacterial time-dependent difference between **3r** concentrations of 2 × and 4 × MIC (80 µM). These concentrations showed a slow reduction in viable cell count over time and a reduction of 3 logs on day 21. Finally, time-kill analysis showed that 1*H*-indole **3r** presented a comparable dynamic behavior to the first-line drug rifampicin [20,21,22].

**Figure 3:**
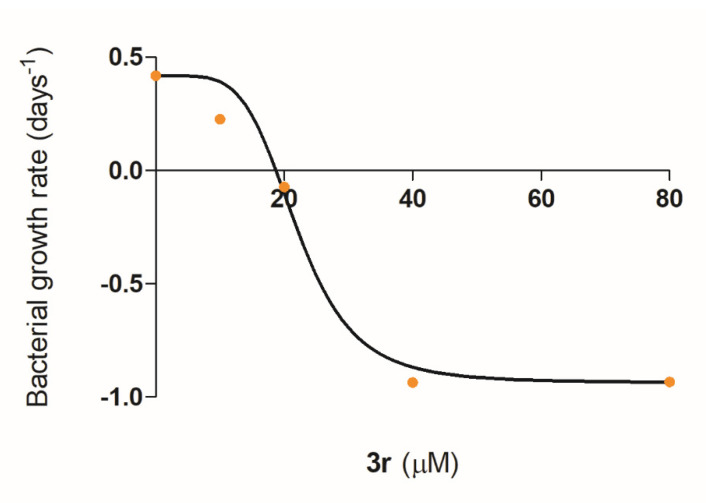
Pharmacodynamic model fitted to estimated growth rates. The points indicate the estimated bacterial growth rates at different concentrations of compound **3r**. The solid line shows the model with the calculated growth rates.

We used the pharmacodynamic model to explore the time-kill curve data from compound **3r**. This model is characterized by four criteria: the maximum rate of bacterial growth in the absence of antimicrobial (ψmax), the minimum rate of bacterial growth in high concentrations of antimicrobial (ψmin), the Hill coefficient (к), and the pharmacodynamic MIC (zMIC) [23,24]. The time-kill curve data provides the bacterial growth rates by fitting a linear regression to the mean colony counts per day for every concentration of 1*H*-indole **3r** (Figure 3). The maximum growth rate in the absence of antimicrobial was ψmax = 0.42 days^−1^. The minimum growth rate was ψmin = −0.94 days^−1^. The Hill coefficient к was 4.93, whereas the estimated zMIC was 18.73 µM. It is noteworthy that the zMIC value agreed with the experimental MIC (19.40 µM). The Hill coefficient (к) describes how the kill rate changes with the change in concentration of the molecule tested around the MIC value. One can observe these changes on the graph as the steepness of the curve [23,24]. Some studies have suggested that a high or low Hill coefficient may be related to the concentration or time-dependent kill kinetics of antibiotics, with a tendency towards higher values for time dependent ones [24]. In addition, it was reported that a median Hill coefficient (к) value of three has been obtained for those drugs endowed with time-dependent kill kinetics [18]. Therefore, the pharmacodynamic model for 1*H*-indole **3r** indicated a strong time-dependent characteristic and a weak concentration-dependent antimycobacterial activity.

In summary, the synthesis and in vitro antimycobacterial activity of a new series of 3-phenyl-1*H*-indoles is shown herein. The highly regioselective synthetic procedure was performed using readily accessible reagents and reactants. In addition, most synthesized compounds showed activity against a drug-susceptible Mtb strain, and the top hit compounds were active against MDR strains without cross-resistance with first-line drugs. Interestingly, one of the compounds was also devoid of apparent toxicity and genotoxicity to mammalian cells. Time-kill and pharmacodynamic model analyses demonstrated that this molecule has bactericidal activity at concentrations close to the MIC (2 × MIC), with a strongly time-dependent behavior. The data presented suggest that this class of molecules may provide candidates for the development of new anti-TB drugs. Hit to lead optimization is underway, and these data will be presented in due course.

## 3. Experimental

### 3.1. Chemistry

Commercially available reactants and solvents were obtained from commercial suppliers and were used without additional purification. The progress of the reaction was monitored using thin-layer chromatography (TLC, Kenilworth, NJ, USA) with Merck TLC Silica gel 60 F254. The melting points were measured using a Microquímica MQAPF-302 apparatus. ^1^H and ^13^C NMR spectra were acquired on an Avance III HD Bruker spectrometer (Bruker Corporation, Fällanden, Switzerland). Chemical shifts (*δ*) were expressed in parts per million (ppm) relative to CDCl_3_, which was used as the solvent, and to TMS, which was used as an internal standard. High-resolution mass spectra (HRMS) were obtained for all compounds on an LTQ Orbitrap Discovery mass spectrometer (Thermo Fisher Scientific, Bremen, Germany). The analyses were performed through the direct infusion of the sample in MeCN/H_2_O (1:1) with 0.1% formic acid, or in MeOH/MeCN (1:1) with 0.025% ammonium hydroxide in a positive-ion or negative-ion mode, respectively, using electrospray ionization (ESI). For elemental composition, calculations were performed using the specific tool included in the Qual Browser module of the Xcalibur software. Compound purity was determined using an Äkta HPLC system (GE Healthcare^®^ Life Sciences, Chicago, IL, USA) equipped with a binary pump, manual injector, and UV detector. Unicorn 5.31 software (Build 743) was used for data acquisition and processing. The HPLC conditions were as follows: RP column, 5 µm Nucleodur C-18 (250 × 4.6 mm); flow rate, 1.5 mL/min; UV detection, 254 nm; 100% water (0.1% acetic acid) was maintained from 0 to 7 min, followed by a linear gradient from 100% water (0.1% acetic acid) to 90% acetonitrile/methanol (1:1, *v*/*v*) from 7 to 15 min (15–30 min) and subsequently returned to 100% water (0.1% acetic acid) in 5 min (30–35 min) and maintained for an additional 10 min (35–45 min). All the evaluated compounds were ≥92% pure.

### 3.2. General Procedure for the Synthesis of 3-Phenyl-1H-indoles *(**3a**–**t**)*

In a screw-cap vial under air, a mixture of Pd(OAc)_2_ (11.3 mg, 0.05 mmol, 5 mol%), dppm (19.2 mg, 0.05 mmol, 5 mol%), LiOH·H_2_O (126 mg, 3.00 mmol), proper aryl-halide (1.20 mmol), and proper indole (1.0 mmol) in degassed H_2_O (2 mL) was vigorously stirred at 110 °C. After 18 h, the reaction mixture was cooled to room temperature and partitioned between 1M HCl (20 mL) and ethyl acetate (20 mL). The layers were separated, and the aqueous layer was further extracted with 4 × 20 mL EtOAc. Combined organic layers were dried over MgSO_4_, filtered, and concentrated under reduced pressure. Purification of the residue by column chromatography using a mixture of hexanes/EtOAc as eluent afforded the desired product.

*3-Phenyl-1H-indole* (**3a**) [13]: Flash column chromatography on a silica gel (hexanes/ethyl acetate, 0 → 90/10) yielded the product as a white solid: 77% yield; mp 82–85 °C. ^1^H-NMR (400 MHz, CDCl_3_) *δ*: 8.20 (1H, bs), 7.95 (1H, d, *J* = 8.0 Hz), 7.67 (2H, dt, *J* = 8.0, 1.5 Hz), 7.48–7.39 (3H, m), 7.36 (1H, d, *J* = 2.5 Hz), 7.32–7.16 (3H, m). ^13^C-NMR (101 MHz, CDCl_3_) *δ*: 136.7, 135.6, 128.7 (2C), 127.5 (2C), 125.9, 125.8, 122.4, 121.7, 120.3, 119.8, 118.4, 111.4. HRMS (ESI): *m*/*z* calc. for C_14_H_10_N [M − H]^−^: 192.0819; obt.: 192.0834.

*3-(p-Tolyl)-1H-indole* (**3b**) [25,26]: Flash column chromatography on a silica gel (petroleum ether/ethyl acetate, 0 → 95/5) yielded the product as a pale yellow solid: 30% yield; mp 73–75 °C. ^1^H-NMR (400 MHz, CDCl_3_) *δ*: 7.91 (1H, d, *J* = 7.5 Hz), 7.78 (1H, bs), 7.52 (2H, d, *J* = 8.1 Hz), 7.18–7.13 (5H, m), 7.09 (1H, d, *J* = 2.5 Hz), 2.36 (3H, s). ^13^C-NMR (101 MHz, CDCl_3_) *δ*: 136.8, 135.7, 132.8, 129.7 (2C), 127.5 (2C), 125.9, 122.4, 121.8, 120.4, 119.9, 118.2, 111.6, 21.3. HRMS (ESI): *m*/*z* calc. for C_15_H_12_N [M − H]^−^: 206.0975; obt.: 206.0989.

*3-(4-Methoxyphenyl)-1H-indole* (**3c**) [13]: Flash column chromatography on a silica gel (petroleum ether/ethyl acetate, 0 → 95/5) yielded the product as a pale-yellow solid: 68% yield; mp 126–129 °C. ^1^H-NMR (400 MHz, CDCl_3_) *δ*: 8.18 (1H, bs), 7.95 (1H, d, *J* = 7.8 Hz), 7.65–7.62 (2H, m), 7.44 (1H, d, *J* = 7.9 Hz), 7.31–7.21 (3H, m), 7.07–7.04 (2H, m), 3.91 (3H, s). ^13^C-NMR (101 MHz, CDCl_3_) *δ*: 158.2, 136.6, 128.7 (2C), 128.6, 128.2, 125.9, 122.3, 121.2, 120.2, 119.8, 118.1, 114.3, 111.4, 55.4. HRMS (ESI): *m/z* calc. for C_15_H_12_NO [M − H]^−^: 222.0924; obt.: 222.0945.

*3-(4-Fluorophenyl)-1H-indole* (**3d**) [27]: Flash column chromatography on a silica gel (hexanes/ethyl acetate, 0 → 90/10) yielded the product as a light yellow solid: 35% yield; mp 96–99 °C. ^1^H-NMR (400 MHz, CDCl_3_) *δ*: 8.08 (1H, bs), 7.89–7.83 (1H, m), 7.60–7.56 (2H, m), 7.39–7.35 (1H, m), 7.27–7.17 (3H, m), 7.15–7.09 (2H, m). ^13^C-NMR (101 MHz, CDCl_3_) *δ*: 161.5 (d, *J* = 244.5 Hz), 136.5, 131.5 (d, *J* = 3.2 Hz), 128.9, 128.8, 125.7, 122.5, 121.6, 120.4, 119.5, 117.4, 115.6 (d, 2C, *J* = 21.3 Hz), 111.4. HRMS (ESI): *m/z* calc. for C_14_H_9_FN [M − H]^−^: 210.0725; obt.: 210.0758.

*3-(4-(Trifluoromethyl)phenyl)-1H-indole* (**3e**) [28]: Flash column chromatography on a silica gel (hexanes/ethyl acetate, 95/5 → 80/10) yielded the product as a light yellow solid: 69% yield; mp 129–132 °C. ^1^H-NMR (400 MHz, CDCl_3_) *δ*: 8.32 (1H, bs), 7.98 (1H, d, *J* = 7.9 Hz), 7.82 (2H, d, *J* = 8.1 Hz), 7.73 (2H, d, *J* = 8.4 Hz), 7.48 (1H, d, *J* = 7.7 Hz), 7.44 (1H, d, *J* = 2.6 Hz), 7.35–7.26 (2H, m). ^13^C-NMR (101 MHz, CDCl_3_) δ: 139.4, 136.8, 127.8 (2C), 127.6 (q, *J* = 33.6 Hz), 127.3, 125.7 (q, *J* = 3.8 Hz), 125.5, 124.5 (q, *J* = 271.6 Hz), 122.8, 122.6, 120.9, 119.6, 117.1, 111.6. HRMS (ESI): *m/z* calc. for C_15_H_9_F_3_N [M − H]^−^: 260.0693; obt.: 260.0725.

*3-(4-Methoxyphenyl)-2-methyl-1H-indole* (**3f**): Flash column chromatography on a silica gel (hexanes/ethyl acetate, 90/10 → 80/20) yielded the product as a brown oil: 10% yield. ^1^H-NMR (400 MHz, CDCl_3_) *δ*: 7.80 (1H, s), 7.54 (1H, d, *J* = 7.8 Hz), 7.38–7.31 (2H, m), 7.22 (1H, dd, *J* = 8.0, 1.1 Hz), 7.11–6.99 (2H, m), 6.96–6.91 (2H, m), 3.79 (3H, d, *J* = 0.7 Hz), 2.38 (3H, s). ^13^C-NMR (101 MHz, CDCl_3_) *δ*: 157.9, 135.2, 131.0, 130.5, 130.4, 128.0, 127.8, 121.4, 119.8, 118.8, 118.7, 114.0, 113.9, 110.2, 55.4, 12.4. HRMS (ESI): *m/z* calc. for C_16_H_14_NO [M − H]^−^: 236.1087; obt.: 236.1070.

*3-(4-Fluorophenyl)-2-methyl-1H-indole* (**3g**): Flash column chromatography on a silica gel (hexanes/ethyl acetate, 90/10→ 80/20) yielded the product as a brown solid: 10% yield; mp 95–98 °C. ^1^H-NMR (400 MHz, CDCl_3_) δ: 7.87 (1H, s), 7.54–7.49 (1H, m), 7.40–7.34 (2H, m), 7.28–7.21 (1H, m), 7.13–7.01 (4H, m), 2.39 (3H, s). ^13^C-NMR (101 MHz, CDCl_3_) *δ*: 161.3 (d, *J* = 244.6 Hz), 135.1, 131.3 (2C), 130.8 (2C), 127.8, 121.6, 120.0, 118.5 (d, *J* = 9.0 Hz), 115.4 (d, *J* = 7.5 Hz), 115.2 (d, *J* = 7.4 Hz), 113.6, 110.3, 12.4. HRMS (ESI): *m/z* calc. for C_15_H_11_NF [M − H]^−^: 224.0887; obt.: 224.0870.

*3-(4-(Trifluoromethyl)phenyl)-2-methyl-1H-indole* (**3h**)*:* Flash column chromatography on a silica gel (hexanes/ethyl acetate, 90/10 → 80/20) yielded the product as a pale yellow solid: 16% yield; mp 128–131 °C. ^1^H-NMR (400 MHz, CDCl_3_) *δ*: 7.90 (1H, s), 7.65–7.60 (2H, m), 7.58–7.50 (3H, m), 7.27–7.22 (1H, m), 7.14–7.03 (2H, m), 2.41 (3H, s). ^13^C-NMR (101 MHz, CDCl_3_) *δ*: 139.4, 135.2, 132.2, 129.4, 127.7 (q, 2C, *J* = 32.2 Hz), 127.4, 125.4 (2C), 124.5 (q, *J* = 271.8 Hz), 121.9, 120.6, 118.5 (q, *J* = 8.5 Hz), 113.4, 110.5, 12.5. HRMS (ESI): *m/z* calc. for C_16_H_11_NF_3_ [M − H]^−^: 274.0858; obt.: 274.0838.

*5-Methyl-3-(4-fluorophenyl)-1H-indole* (**3i**) [29]: Flash column chromatography on a silica gel (hexanes/ethyl acetate, 0 → 90/10) yielded the product as a light yellow solid: 20% yield; mp 121–123 °C. ^1^H-NMR (400 MHz, CDCl_3_) *δ*: 8.12 (1H, s), 7.64 (1H, s), 7.61–7.54 (2H, m), 7.29 (1H, d, *J* = 8.3 Hz), 7.23 (1H, d, *J* = 2.5 Hz), 7.15–7.05 (3H, m), 2.47 (3H, s). ^13^C-NMR (101 MHz, CDCl_3_) *δ*: 161.4 (d, *J* = 244.3 Hz), 134.9, 131.7 (d, *J* = 3.3 Hz), 129.7, 128.9, 128.8, 125.9, 124.1, 121.7 (2C), 119.1, 116.9, 115.5 (d, *J* = 21.3 Hz), 111.1, 21.6. HRMS (ESI): *m/z* calc. for C_15_H_11_NF [M − H]^−^: 224.0884; obt.: 224.0870.

*5-Methyl-3-(4-(trifluoromethyl)phenyl)-1H-indole* (**3j**) [30]: Flash column chromatography on a silica gel (hexanes/ethyl acetate, 0 → 90/10) yielded the product as a light yellow oil: 16% yield. ^1^H-NMR (400 MHz, CDCl_3_) *δ*: 8.32 (1H, s), 7.75 (2H, dt, *J* = 7.9, 0.8 Hz), 7.69 (3H, ddt, *J* = 8.8, 7.3, 0.8 Hz), 7.37 (1H, d, *J* = 2.6 Hz), 7.33 (1H, dd, *J* = 8.3, 0.7 Hz), 7.10 (1H, dd, *J* = 8.3, 1.6 Hz), 2.49 (3H, s). ^13^C-NMR (101 MHz, CDCl_3_) *δ*: 139.5 (d, *J* = 1.4 Hz), 135.1, 130.1, 127.6 (q, 2C, *J* = 32.2 Hz), 127.3 (2C), 125.6 (q, 2C, *J* = 3.9 Hz), 124.5 (q, *J* = 271.7 Hz), 124.4, 122.7, 119.1, 116.6, 111.3, 21.6. HRMS (ESI): *m/z* calc. for C_16_H_11_NF_3_ [M − H]^−^: 274.0854; obt.: 274.0838.

*5-Methoxy-3-(p-tolyl)-1H-indole* (**3k**) [26]: Flash column chromatography on a silica gel (hexanes/ethyl acetate, 0 → 90/10) yielded the product as a pale yellow solid: 58% yield; mp 141–144 °C. ^1^H-NMR (400 MHz, CDCl_3_) *δ*: 8.05 (1H, bs), 7.53 (2H, d, *J* = 8.0 Hz), 7.36 (1H, d, *J* = 2.4 Hz), 7.30–7.23 (4H, m), 6.90 (1H, dd, *J* = 8.8, 2.4 Hz), 3.85 (3H, s), 2.40 (3H, s). ^13^C-NMR (101 MHz, CDCl_3_) *δ*: 154.7, 135.5, 132.7, 131.8, 129.4 (2C), 127.3 (2C), 126.3, 122.3, 118.1, 112.6, 112.0, 101.8, 56.0, 21.1. HRMS (ESI): *m/z* calc. for C_16_H_16_NO [M + H]^+^: 238.1226; obt.: 238.1251.

*5-Methoxy-3-(4-methoxyphenyl)-1H-indole* (**3l**) [27]: Flash column chromatography on a silica gel (hexanes/ethyl acetate, 0 → 90/10) yielded the product as a pale yellow solid: 26% yield, mp 72–75 °C. ^1^H-NMR (400 MHz, CDCl_3_) *δ*: 8.13 (1H, s), 7.60–7.52 (2H, m), 7.35–7.28 (3H, m), 7.03–6.98 (2H, m), 6.91 (1H, dd, *J* = 8.8, 2.5 Hz), 3.86 (6H, s). ^13^C-NMR (101 MHz, CDCl_3_) δ: 154.7 (2C), 131.8, 128.6 (3C), 122.0 (3C), 114.3 (2C), 112.6, 112.0, 101.6, 56.0, 55.4. HRMS (ESI): *m/z* calc. for C_16_H_16_NO_2_ [M + H]^+^: 254.1176; obt.: 254.1177.

*5-Methoxy-3-(4-fluorophenyl)-1H-indole* (**3m**): Flash column chromatography on a silica gel (hexanes/ethyl acetate, 95/5 → 90/10) yielded the product as a yellow oil that solidified upon cooling: 53% yield. ^1^H-NMR (400 MHz, CDCl_3_) *δ*: 8.01 (1H, s), 7.49–7.42 (2H, m), 7.21 (1H, dd, *J* = 2.4, 0.7 Hz), 7.15 (1H, dd, *J* = 8.8, 0.6 Hz), 7.12–7.09 (1H, m), 7.07–6.99 (2H, m), 6.82 (1H, ddd, *J* = 8.8, 2.4, 0.5 Hz), 3.75 (3H, s). ^13^C-NMR (101 MHz, CDCl_3_) *δ*: 161.3 (d, *J* = 244.4 Hz), 154.7, 131.7, 128.8, 128.6, 126.0, 122.5, 122.4, 117.0, 115.6 (d, 2C, *J* = 21.2 Hz), 112.6, 112.2, 101.3, 56.0. HRMS (ESI): *m/z* calc. for C_15_H_11_NOF [M − H]^−^: 240.0832; obt.: 240.0819.

*5-Methoxy-3-(4-(trifluoromethyl)phenyl)-1H-indole* (**3n**)*:* Flash column chromatography on a silica gel (hexanes/ethyl acetate, 0 → 90/10) yielded the product as a dark yellow solid: 43% yield; mp 90–93 °C. ^1^H-NMR (400 MHz, CDCl_3_) *δ*: 8.23 (1H, bs), 7.74 (2H, d, *J* = 8.6 Hz), 7.68 (2H, d, *J* = 8.4 Hz), 7.37 (2H, q, *J* = 4.3 Hz), 7.33 (1H, d, *J* = 8.8 Hz), 6.94 (1H, q, *J* = 3.7 Hz), 3.87 (1H, s). ^13^C-NMR (101 MHz, CDCl_3_) *δ*: 155.1, 139.5 (d, *J* = 1.2 Hz), 131.9, 127.8 (q, 2C, *J* = 33.0 Hz), 127.2, 125.9, 125.7 (q, 2C, *J* = 3.8 Hz), 124.5 (q, *J* = 270.7 Hz), 123.3, 116.9, 113.0, 112.3, 101.6, 56.0. HRMS (ESI): *m/z* calc. for C_16_H_11_NOF_3_ [M − H]^−^: 290.0798; obt.: 290.0787.

*5-Fluoro-3-(4-fluorophenyl)-1H-indole* (**3o**) [29]: The general procedure was followed although the temperature was 90 °C. Flash column chromatography on a silica gel (hexanes/ethyl acetate, 90/10 → 70/30) yielded the product as a brown oil: 10% yield. ^1^H-NMR (400 MHz, CDCl_3_) *δ*: 8.28 (1H, s), 7.60–7.50 (2H, m), 7.50 (1H, dd, *J* = 9.9, 2.5 Hz), 7.33 (2H, dd, *J* = 8.6, 3.7 Hz), 7.19–7.08 (2H, m), 7.00 (1H, td, *J* = 9.0, 2.5 Hz). ^13^C-NMR (101 MHz, CDCl_3_) *δ*: 161.6 (d, *J* = 245.0 Hz), 158.5 (d, *J* = 235.1 Hz), 133.1, 131.1 (d, *J* = 3.3 Hz), 128.8 (d, 2C, *J* = 7.8 Hz), 126.1 (d, *J* = 9.9 Hz), 123.3, 117.7 (d, *J* = 4.6 Hz), 115.7 (d, 2C, *J* = 21.5 Hz), 112.1 (d, *J* = 9.7 Hz), 110.9 (d, *J* = 26.4 Hz), 104.5 (d, *J* = 24.2 Hz). HRMS (ESI): *m/z* calc. for C_14_H_8_F_2_N [M − H]^−^: 228.0630; obt.: 228.0652.

*5-Fluoro-3-(4-(trifluoromethyl)phenyl)-1H-indole* (**3p**): The general procedure was followed although the reaction temperature was 90 °C. Flash column chromatography on a silica gel (hexanes/ethyl acetate, 95/5 → 90/10) yielded the product as a brown oil: 35% yield. ^1^H-NMR (400 MHz, CDCl_3_) *δ*: 8.47 (1H, s), 7.73–7.63 (4H, m), 7.55 (1H, dd, *J* = 9.9, 2.5 Hz), 7.44 (1H, d, *J* = 2.7 Hz), 7.34 (1H, dd, *J* = 8.9, 4.4 Hz), 7.01 (1H, td, *J* = 9.0, 2.5 Hz). ^13^C-NMR (101 MHz, CDCl_3_) *δ*: 158.6 (d, *J* = 235.6 Hz), 138.9 (d, *J* = 1.5 Hz), 133.3, 127.9 (q, *J* = 32.4 Hz), 127.1 (2C), 125.8 (q, *J* = 3.8 Hz), 124.2, 123.1, 121.2 (q, *J* = 170.9 Hz), 117.2 (d, *J* = 4.7 Hz), 112.3 (d, *J* = 9.7 Hz), 111.2 (d, *J* = 26.4 Hz), 104.6 (d, *J* = 24.3 Hz). HRMS (ESI): *m/z* calc. for C_15_H_8_NF_4_ [M − H]^−^: 278.0611; obt.: 278.0587.

*5-Chloro-3-phenyl-1H-indole* (**3q**) [31]: Flash column chromatography on a silica gel (hexanes/ethyl acetate, 0 → 93/7) yielded the product as a light yellow solid: 67% yield; mp 87–90 °C. ^1^H-NMR (400 MHz, CDCl_3_) *δ*: 8.24 (1H, bs), 7.94 (1H, d, *J* = 1.5 Hz), 7.67–7.65 (2H, m), 7.50 (2H, t, *J* = 7.7 Hz), 7.40–7.31 (3H, m), 7.24 (1H, dd, *J* = 1.9, 8.6 Hz). ^13^C-NMR (101 MHz, CDCl_3_) *δ*: 135.0, 134.8, 128.9 (2C), 127.5 (2C), 126.9, 126.4, 126.2, 123.0, 122.8, 119.4, 118.3, 112.4. HRMS (ESI): *m/z* calc. for C_15_H_9_ClN [M − H]^−^: 226.0429; obt.: 226.0454.

*5-Chloro-3-(4-methoxyphenyl)-1H-indole* (**3r**) [31]: Flash column chromatography on a silica gel (hexanes/ethyl acetate, 0 → 93/7) yielded the product as brown oil: 10% yield. ^1^H-NMR (400 MHz, CDCl_3_) *δ*: 8.27 (1H, bs), 7.87–7.81 (1H, m), 7.57–7.48 (2H, m), 7.35–7.27 (2H, m), 7.18 (1H dd, *J* = 8.6, 2.0 Hz), 7.03–6.97 (2H, m), 3.86 (3H, s). ^13^C-NMR (101 MHz, CDCl_3_) *δ*: 158.3, 134.9, 132.7, 128.6, 127.4, 127.0, 125.9, 122.6, 122.4, 119.2, 117.9, 114.4 (2C), 112.3, 55.4. HRMS (ESI): *m/z* calc. for C_15_H_11_NOCl [M − H]^−^: 256.0524; obt.: 256.0561.

*5-Chloro-3-(4-fluorophenyl)-1H-indole* (**3s**)*:* Flash column chromatography on a silica gel (hexanes/ethyl acetate, 95/5 → 90/10) yielded the product as yellow oil: 21% yield. ^1^H-NMR (400 MHz, CDCl_3_) *δ*: 8.26 (1H, bs), 7.81 (1H, d, *J* = 1.8 Hz), 7.58–7.50 (2H, m), 7.35–7.29 (2H, m), 7.19 (1H, dt, *J* = 8.7, 1.6 Hz), 7.17–7.10 (2H, m). ^13^CNMR (101 MHz, CDCl_3_) *δ*: 161.6 (d, *J* = 245.2 Hz), 134.9, 130.8 (d, *J* = 3.3 Hz), 128.9 (d, *J* = 7.8 Hz), 126.8, 126.2, 122.8 (2C), 119.0 (2C), 117.3, 115.7 (d, *J* = 21.5 Hz, 2C), 112.4. HRMS (ESI): *m/z* calc. for C_14_H_8_NClF [M − H]^−^: 244.0345; obt.: 244.0324.

*5-Chloro-3-(4-(trifluoromethyl)phenyl)-1H-indole* (**3t**) [29]: Flash column chromatography on a silica gel (hexanes/ethyl acetate, 0 → 93/7) gives the product as a light yellow solid: 30% yield; mp 125–128 °C. ^1^H-NMR (400 MHz, CDCl_3_) *δ*: 8.33 (1H, bs), 7.85 (1H, d, *J* = 1.9 Hz), 7.67 (4H, s), 7.39 (1H, d, *J* = 2.6 Hz), 7.32 (1H, d, *J* = 8.6 Hz), 7.21 (1H, q, *J* = 3.6 Hz). ^13^C-NMR (101 MHz, CDCl_3_) *δ*: 138.5 (1C, d, *J* = 1.1 Hz), 135.0, 128.1 (1C, q, *J* = 32.4 Hz), 127.3 (3C), 126.6, 126.5, 125.8 (2C, q, *J* = 3.8 Hz), 123.8, 123.1, 119.0, 116.8, 112.6. HRMS (ESI): *m/z* calc. for C_15_H_8_ClF_3_N [M − H]^−^: 294.0303; obt.: 294.0326.

### 3.3. Determination of the Minimum Inhibitory Concentration (MIC)

The determination of the MIC was performed in sterile 96-well, clear round-bottom plates using the resazurin reduction microplate assay (REMA), as previously described [9]. Briefly, test compounds **3a**–**t** were solubilized in 100% DMSO (Sigma-Aldrich, St. Louis, MO, USA) to a concentration of 2 mg/mL, and aliquots were kept at −20 °C. On the day of the assay, compound aliquots were thawed at room temperature and diluted in Middlebrook 7H9 liquid medium (Becton Dickinson, BD), containing 10% (*v*/*v*) BBLTM Middlebrook ADC enrichment (albumin, dextrose, and catalase, BD) and 5% (*v*/*v*) DMSO, to concentrations limited by the solubility of each compound. Test compounds were two-fold serially diluted, resulting in a 10-point concentration range. Isoniazid (INH, ACROS) was used as the anti-TB drug control. Compounds had their activity evaluated against the Mtb H37Rv laboratory reference strain (ATCC 27294) and three multidrug resistant clinical isolates of Mtb (PT2, PT12, and PT20), which were isolated from patients in the Lisbon Health Region, Lisbon, Portugal [14]. The MIC was considered as the lowest compound concentration capable of preventing resazurin (Sigma-Aldrich) reduction, which is otherwise marked by a color switch from blue to bright pink. Assays were performed three times for each compound on different dates, and only the most frequent MIC value among the assays was reported in molar concentration (µM).

### 3.4. Cellular Evaluation

Cellular viability determination was conducted after incubation of cells with the **3h**, **3n**, **3r** and **3t** compounds using both the 3-(4,5-dimethylthiazol-2-yl)-2,5-diphenyl-2*H*-tetrazolium bromide (MTT) [15] and neutral red uptake (NRU) [16] methods. HepG2 and Vero cells were grown in Dulbecco’s Modified Eagle Medium (DMEM—Invitrogen, Waltham, MA, USA) supplemented with 10% inactivated fetal bovine serum (Invitrogen), 1% penicillin—streptomycin (Invitrogen), and 0.1% fungizone (Invitrogen). For the MTT and NRU assay, HepG2 (4 × 10^3^ cells/well) and Vero (2 × 10^3^ cells/well) cells were seeded in 96-well culture plates and incubated for 24 h. 1*H*-Indoles were diluted at concentrations of 30 µM using DMSO 0.5% and were incubated with the cell lines for 72 h at 37 °C. Importantly, 30 µM was the maximum concentration allowed by the solubility of the molecules. For the MTT assay, after incubation for 72 h at 37 °C under 5% CO_2_, the cultures were incubated with MTT solution (5 mg/mL) for 4 h. The formazan crystals were dissolved with 100 μL of DMSO. The absorbance was measured at 595 nm using an EZ Read 400 microplate reader (Biochrom, Holliston, MA, USA). The mean absorbance of negative control wells was set as 100% viability, and the values of treated cells were calculated as the percentage of vehicle control (0.5% DMSO). The precipitated purple formazan crystals were directly proportional to the number of live cells with active mitochondria. For the NRU assay, after 72 h of cell incubation PBS was used to wash the cells and 200 μL of neutral red dye solution (25 μg/mL, Sigma) prepared in serum-free medium was added to the plate and incubated for 3 h at 37 °C under 5% CO_2_. Cells were washed with PBS followed by the addition of 100 μL of a desorb solution (ethanol/acetic acid/water, 50:1:49) for 30 min with gently shaking to extract neutral red dye from the viable cells. Absorbance was measured at 562 nm (EZ Read 400 microplate reader (Biochrom, Holliston, MA, USA)), and the cell viability was expressed as a percentage, considering the vehicle control cell (0.5% DMSO) as 100% cell viability.

### 3.5. Genotoxicity

The alkaline comet assay [17] was performed using HepG2 cells. The cells were seeded at 8 × 10^4^ cells/well in a 24-well culture plate for 24 h before treatment. After this time, compound **3r** was added at concentrations of 20 μM and 30 μM, and the plate was incubated for an additional 24 h. Cells were mixed with low-melting point agarose and placed on a microscope slide precovered with normal agarose, and placed at 4 °C for 10 min for total agarose solidification. The microscope slides were then exposed to a lysis solution (2.5 M NaCl, 100 mM Na_2_EDTA, 10 mM Tris with 1% Triton X-100, and 10% DMSO) for 48 h. The slides were washed with PBS and then exposed to alkali conditions (300 mM NaOH, 1 mM ethylenedinitrilotetraacetic acid, pH > 13) at 4 °C for 20 min in order to allow the DNA to unfold and the expression of alkali-labile sites to occur. Electrophoresis was then performed for 20 min at 25 V and 300 mA. Subsequently, the slides were neutralized, fixed, and stained using silver nitrate staining. After they were completely dry, the cells were observed on a microscope. Incubation with methyl methanesulfonate (MMS) at 100 µM was used as a positive control group. Overall, 100 cells from each slide, and two slides per treatment, were selected randomly for the analysis and for quantifying DNA damage. Slides were visually scored according to the size and amount of DNA present in the tail. Separately, each cell was given an arbitrary value of 0 (undamaged) to 4 (maximum damage). The damage score was thus attributed to each slide and ranged from 0 (undamaged: 100 cells × 0) to 400 (maximum damage: 100 cells × 4). Finally, damage score was expressed as the mean with standard error from two independent experiments.

### 3.6. Time-Kill Curves

To observe the kill kinetics and the MBC of compound **3r** against the Mtb H37Rv, time-killing curves were performed as previously described [22]. First, an actively growing Mtb culture (OD600 ≈ 0.6–0.8) was diluted to a theoretical OD of 0.1 in 7H9 broth supplemented with 10% ADC, 0.05% Tween-80, and 0.2% glycerol. Next, the former mycobacterial suspension was further diluted (1:500) in 7H9 broth to reach a cell density of approximately 10^5^ CFU/mL. This second mycobacterial suspension was equally divided (5 mL) in 50 mL conical tubes (Corning^®^, New York, NY, USA), each containing different concentrations of compound **3r** (0.5, 1.0, 2.0, or 4.0 × the MIC value in liquid medium) in triplicate. Aliquots (0.1 mL) were taken from this second mycobacterial suspension on day zero, in triplicate, serially diluted in sterile 0.9% saline solution (10^−1^, 10^−2^ and 10^−3^), and plated onto nonselective 7H10 agar plates, supplemented with 10% OADC and 0.5% glycerol, to determine the initial inoculum. The final concentration of DMSO was adjusted to 2.5% in all tubes. A tube containing DMSO only (no compound) was included as a growth control. The cultures were incubated under shaking (37 °C, 100 rpm) for 21 days. Aliquots (0.1 mL) were taken from each tube on days 3, 7, 14 and 21 after inoculation, serially diluted in sterile 0.9% saline solution, and three dilutions were chosen to be spread (0.1 mL) onto 7H10 agar plates. The plates were incubated at 37 °C for 2–3 weeks until colonies were visible for CFU counting. The CFU/mL values were converted to the logarithmic scale (log_10_) and plotted as a function of the exposure time in days. A lower limit of detection of 200 CFU/mL for undiluted samples was fixed for this experiment. The MBC value was considered as the lowest compound concentration that resulted in 3 logs kill of Mtb over 21 days [22].

### 3.7. Pharmacodynamic Model

The maximum and minimum bacterial growth rates (ψ) were obtained from the coefficient of a linear regression from the colony count of viable bacteria (CFU/mL) over 21 days of the time-kill curve of compound **3r**. The Hill coefficient and zMIC results were obtained using the least squares method, using an algorithm to minimize the differences between the experimental values and the estimated values [23,24].

### 3.8. Statistical Analysis

Data were expressed as the mean ± standard error of the mean. Statistical analysis was performed by one-way analysis of variance followed by Dunnett’s post-test, using GraphPadPrism 5.0 software.

## Figures and Tables

**Table 1:** In vitro activity of the selected 1*H*-indoles against *M. tuberculosis* H37Rv and MDR strains and an evaluation of the viability of HepG2 and Vero cells.

Compound	H37Rv (μM)	PT2 (μM)	PT12 (μM)	PT20 (μM)	CC_50_ ^a^ HepG2 (µM)	CC_50_ ^a^ Vero (µM)
**3h**	18.2	4.5	9.1	9.1	<30	<30
**3n**	17.2	17.2	17.2	17.2	<30	<30
**3r**	19.4	9.7	19.4	19.4	>30	>30
**3t**	8.4	8.4	8.4	8.4	<30	<30
**INH**	2.3	291.7	36.4	145.8	-	-

^a^ Concentration that inhibits 50% of cellular viability determined by MTT and Neutral Red assays. INH, Isoniazid.

## Data Availability

The data presented in this study are available in Supplementary Materials.

## Supplementary Materials

The following are available online. Figures S1–S40: ^1^H and ^13^C spectra for compounds **3a–3t**.

## Sample Availability

Samples of the synthesized compounds are available from the authors.
