# Supplementary material for: Synthesis and Antimycobacterial Activity of 3-Phenyl-1H-indoles
Source: Molecules. 2021 Aug 25;26(17):5148. doi: 10.3390/molecules26175148 (PMC8433792; doi:10.3390/molecules26175148)
Supplement: Supplementary file 1 [file molecules-26-05148-s001.zip › molecules-1336903-supplementary.pdf]

Synthesis and antimycobacterial activity of 3-phenyl-1*H*-indoles

Renata Jardim Etchart, Raoní S. Rambo, Bruno Lopes Abbadi, Nathalia Sperotto, Christiano Ev Neves, Fernanda Fries Silva, Maiele Dornelles, Lovaine Duarte, Fernanda Souza Macchi, Marcia Alberton Perelló, Rogério Vescia Lourega, Cristiano Valim Bizarro, Luiz Augusto Basso, Pablo Machado,*

<u>Table of contents:</u>

NMR Spectra for compounds **3a-3t**…...…………………………….…………….……..............2-21

NMR Spectra for compounds 3a-3t:

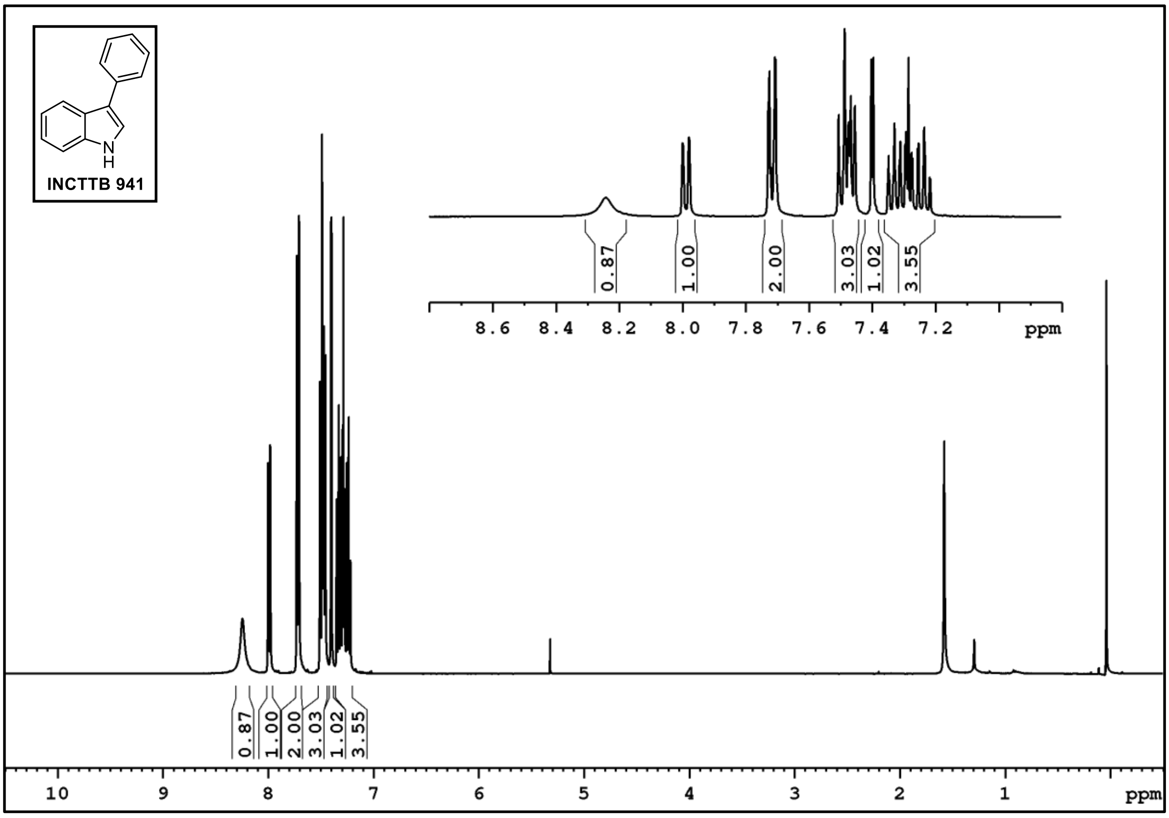

Figure S1 – ^{1}H NMR spectrum of compound **3a**.

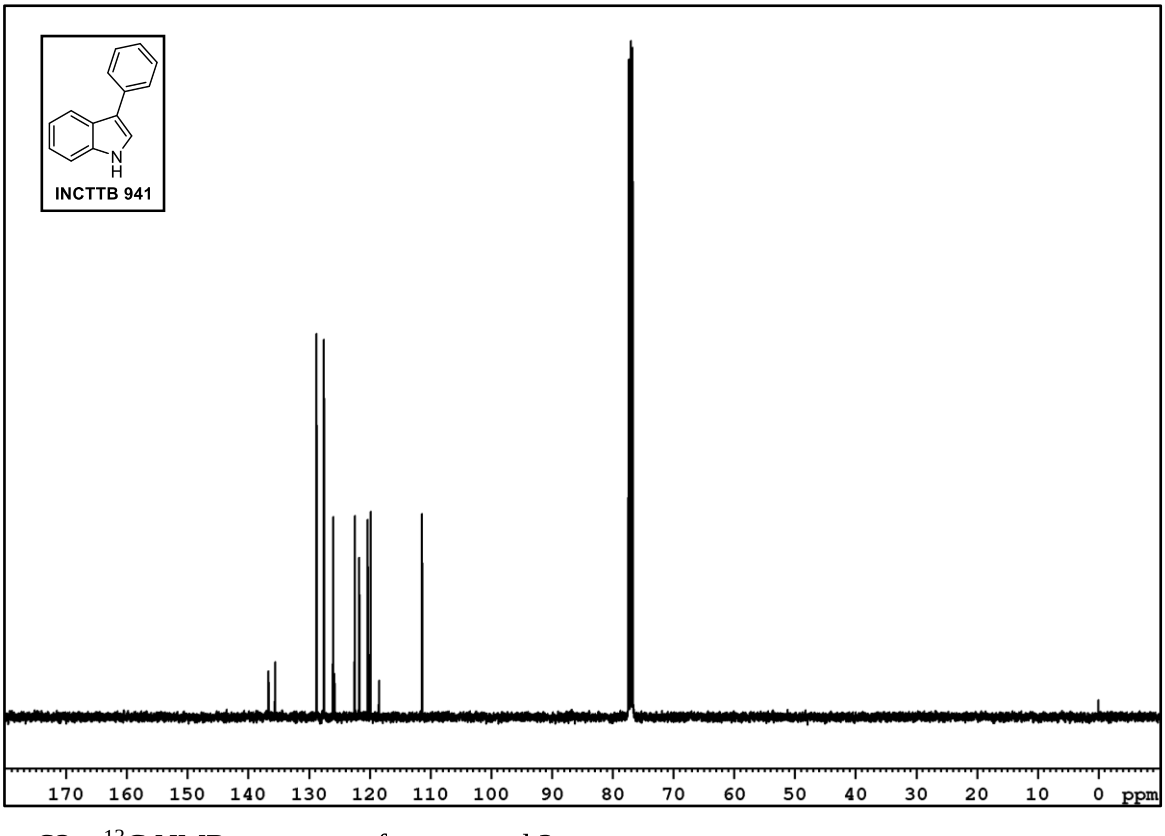

Figure S2 – ^{13}C NMR spectrum of compound **3a**.

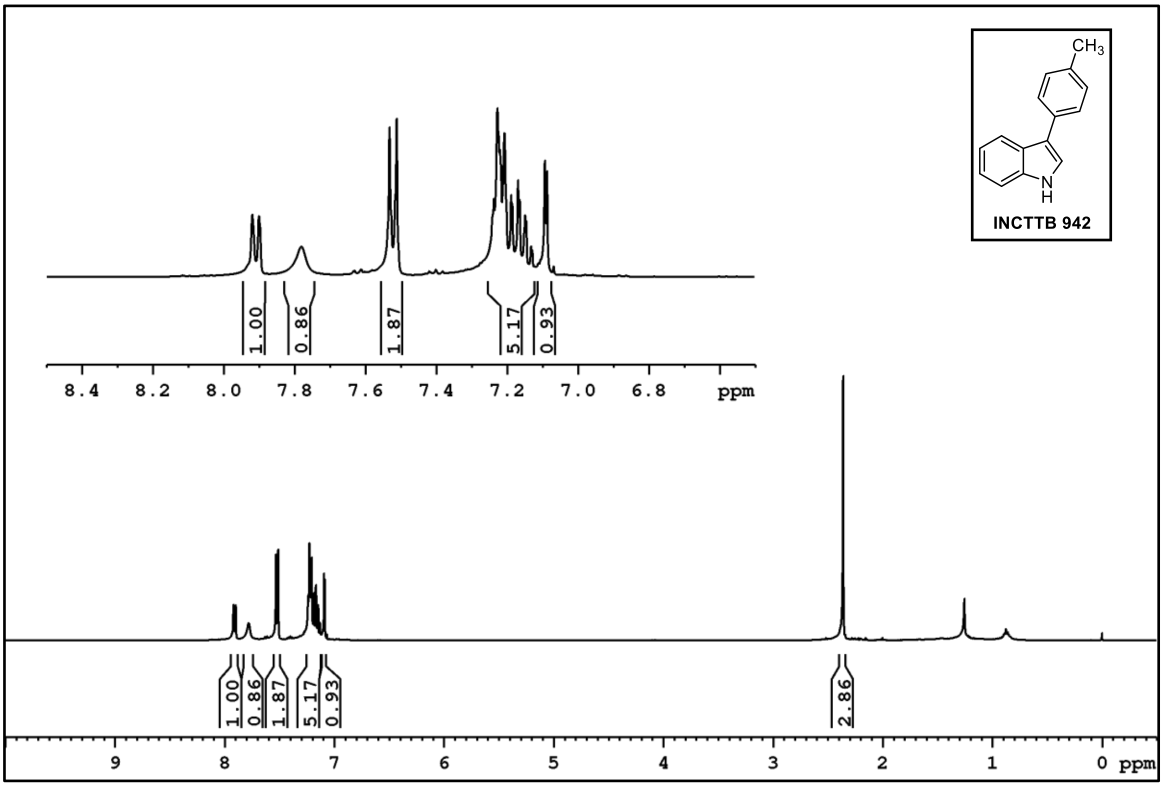

Figure S3 – ^{1}H NMR spectrum of compound **3b**.

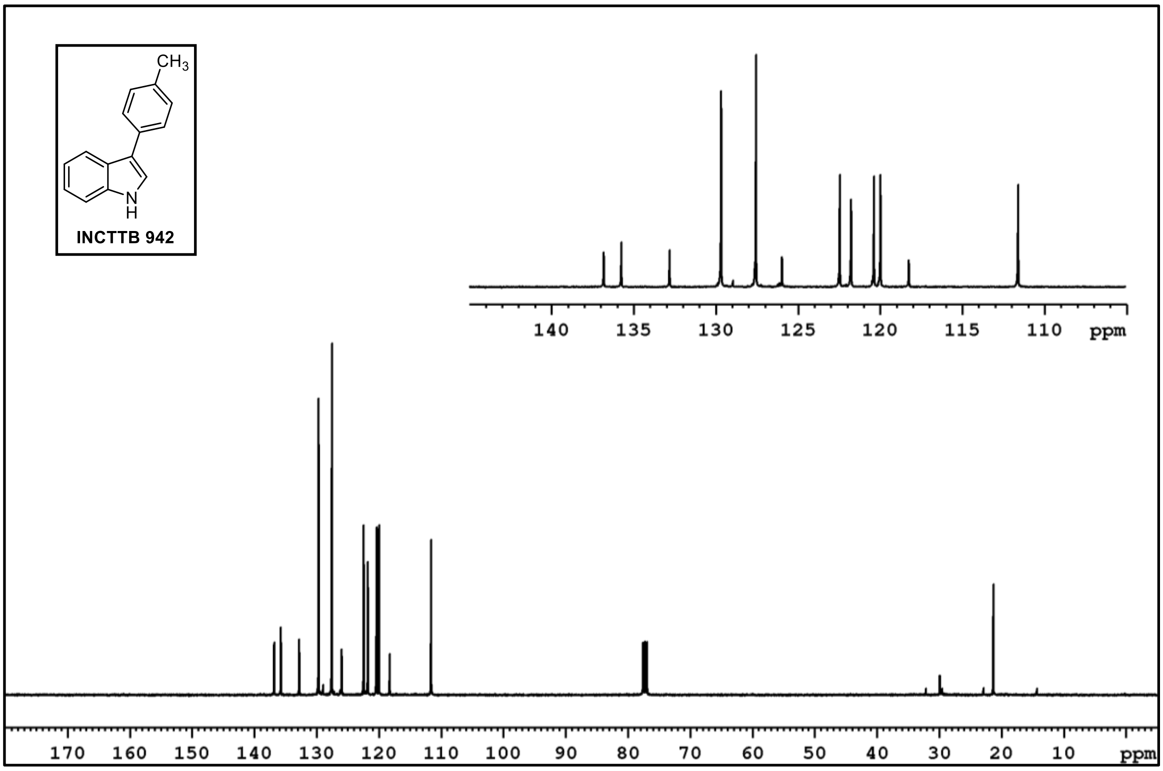

Figure S4 – ^{13}C NMR spectrum of compound **3b**.

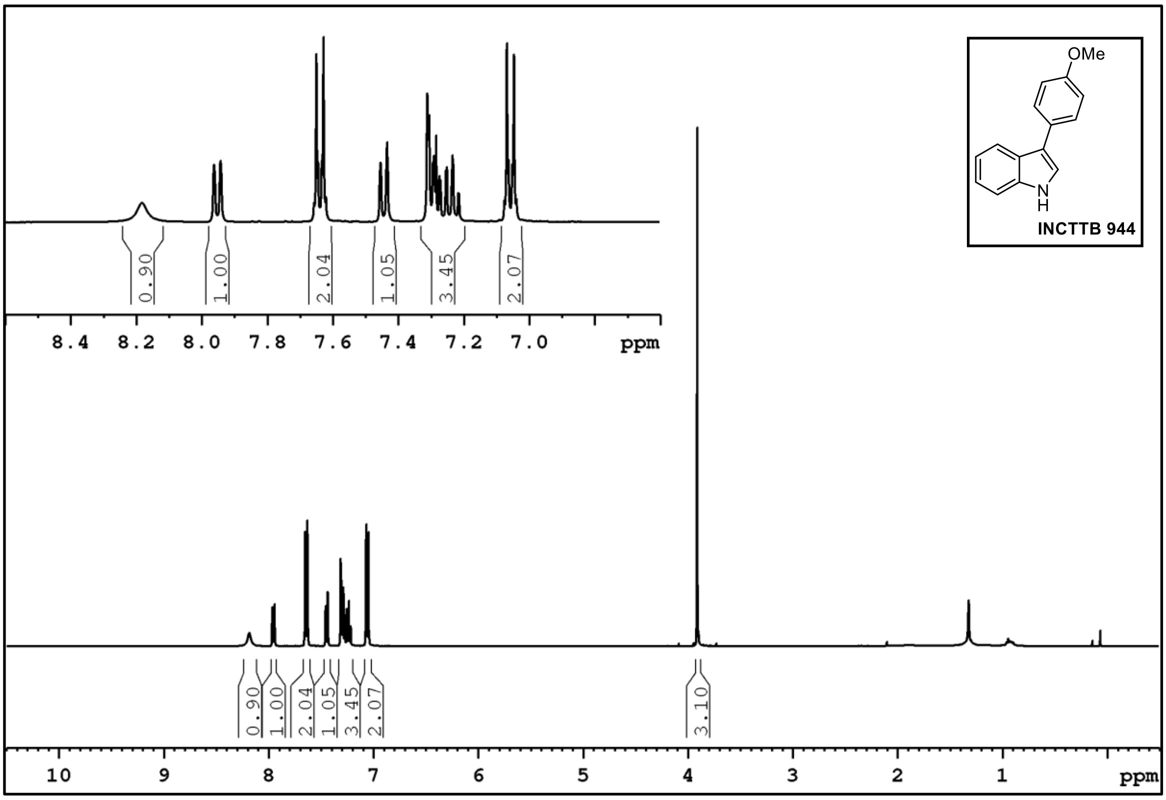

Figure S5 – ^{1}H NMR spectrum of compound **3c**.

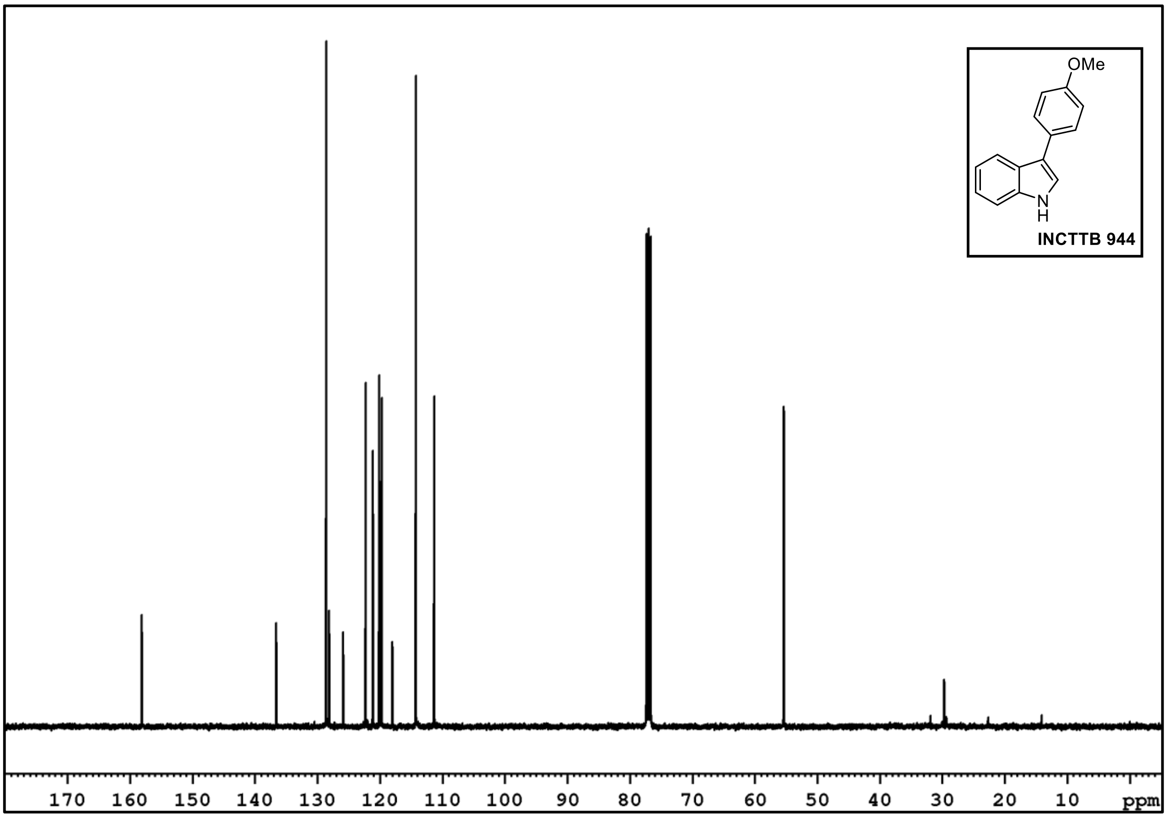

Figure S6 – ^{13}C NMR spectrum of compound **3c**.

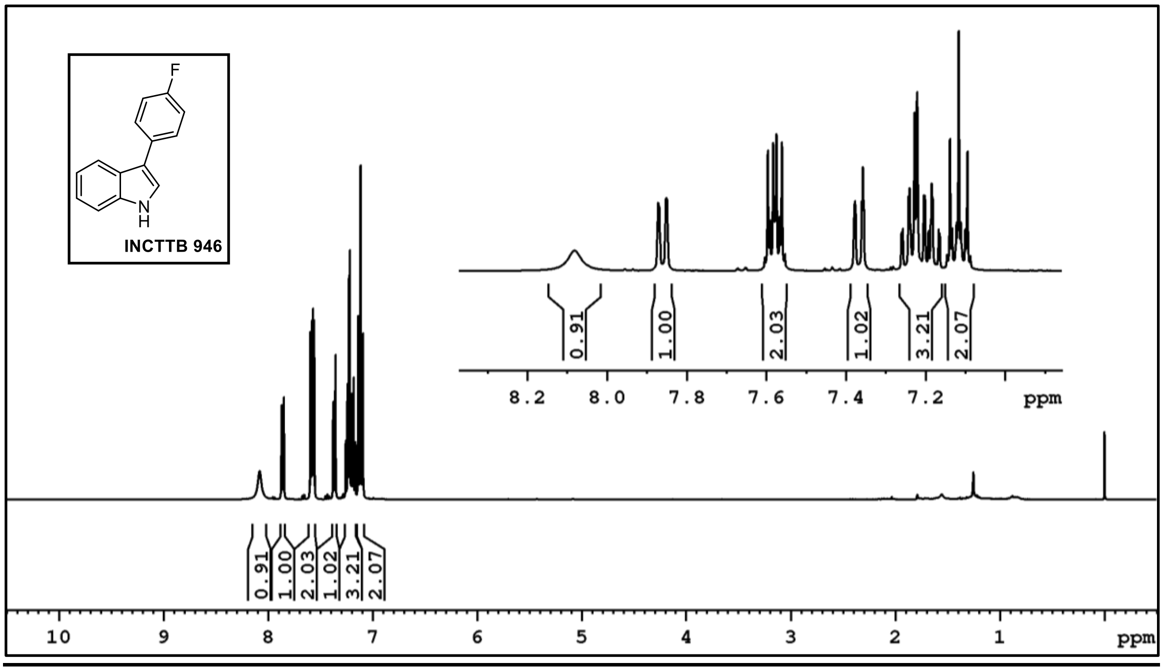

Figure S7 – ^{1}H NMR spectrum of compound **3d**.

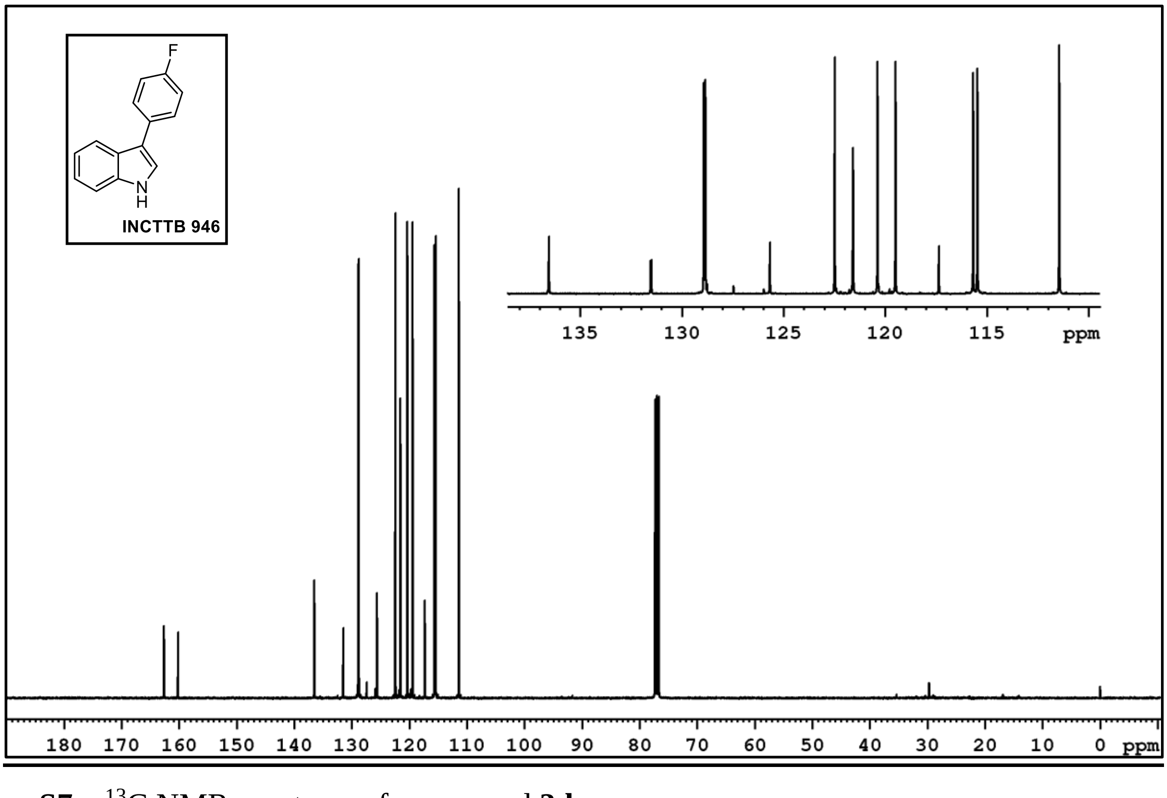

Figure S7 – ^{13}C NMR spectrum of compound **3d**.

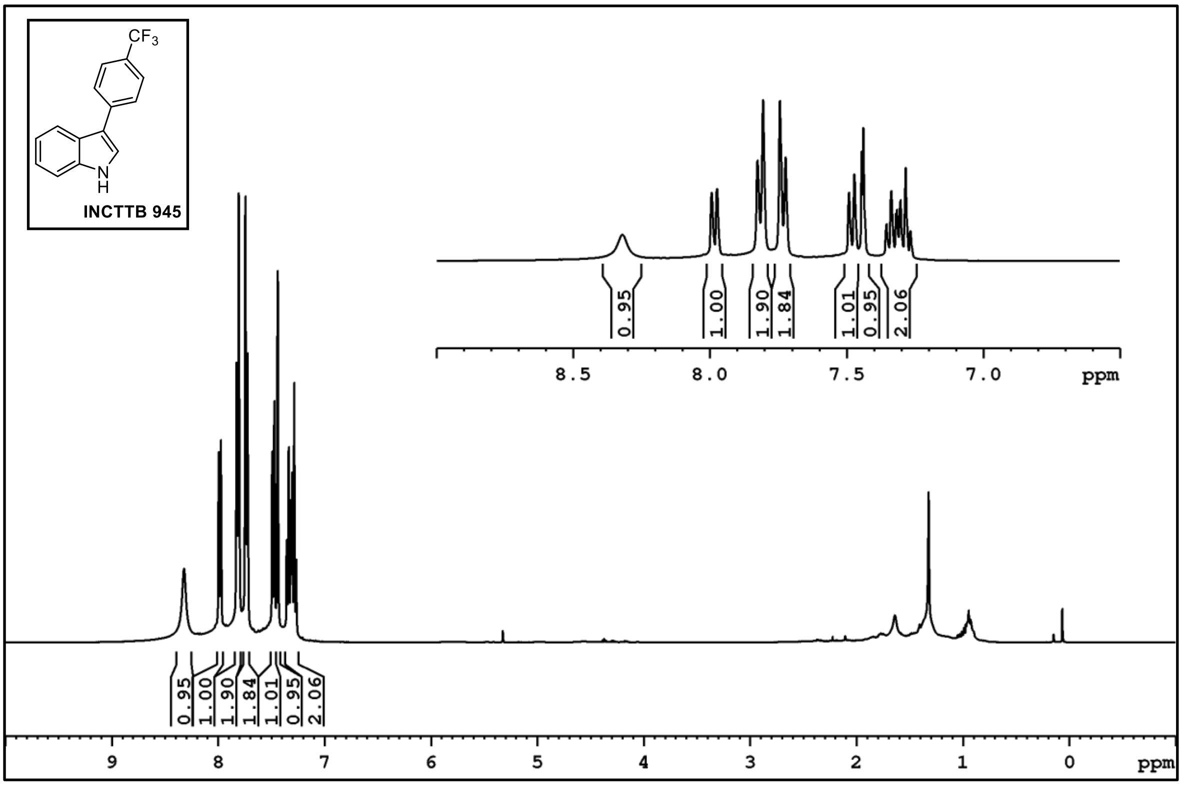

Figure S9 – ^{1}H NMR spectrum of compound **3e**.

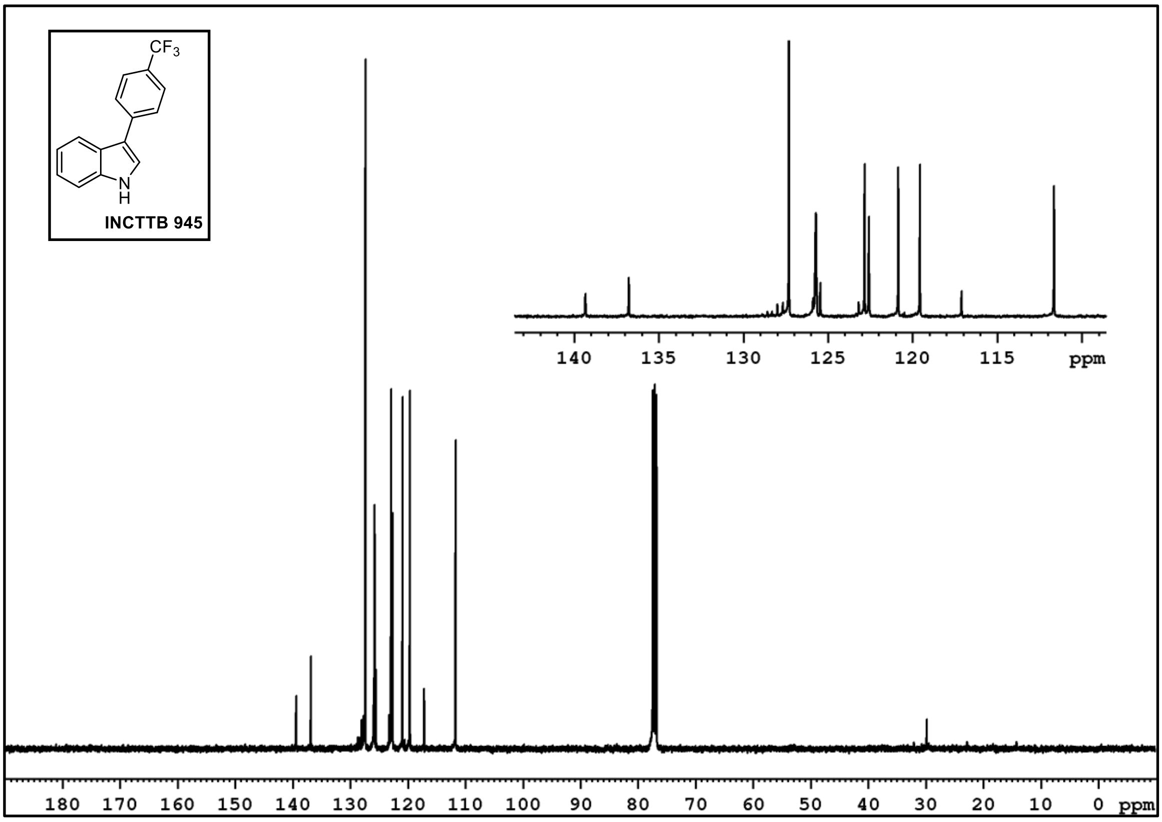

Figure S10 – ^{13}C NMR spectrum of compound **3e**.

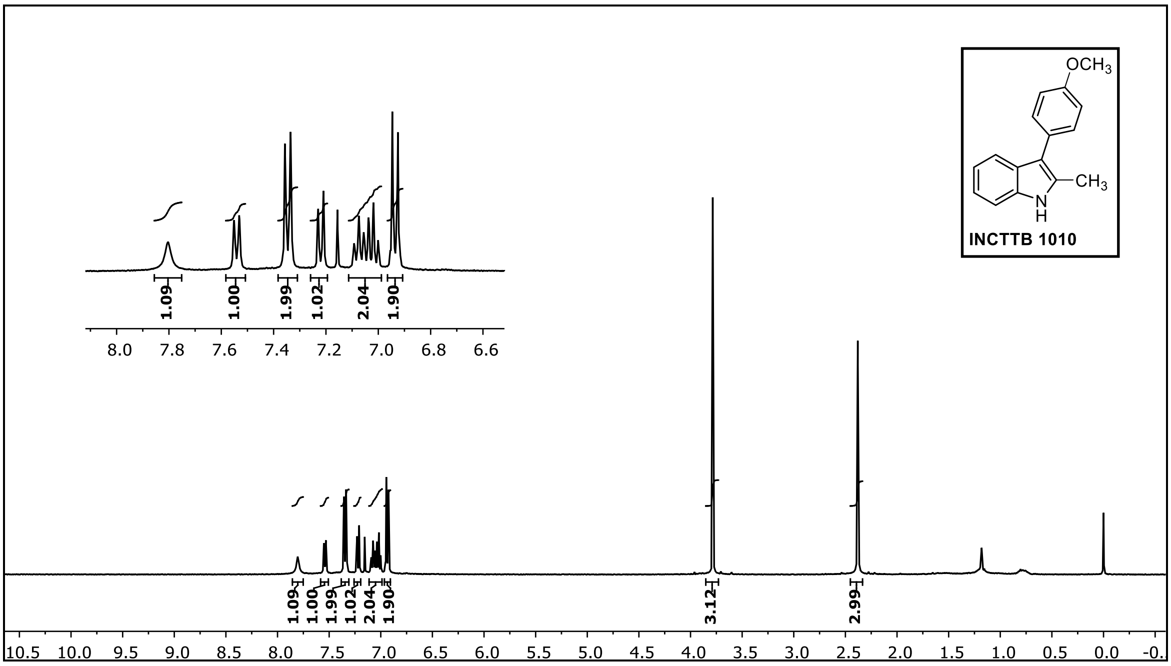

Figure S11 – ^{1}H NMR spectrum of compound **3f**.

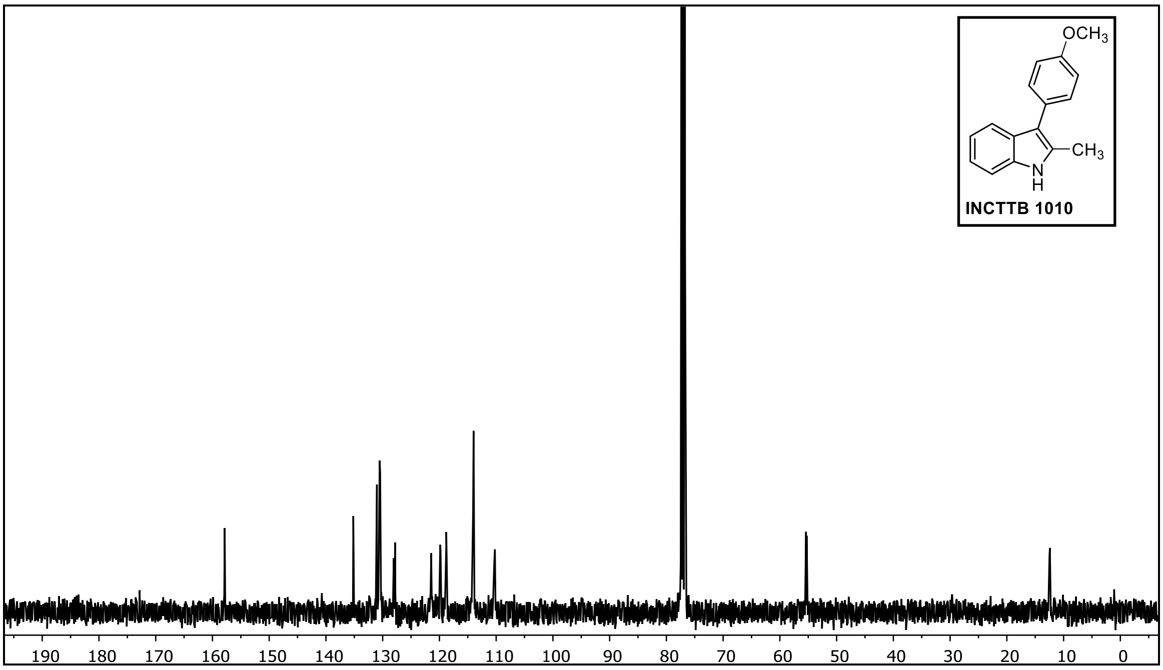

Figure S12 – ^{13}C NMR spectrum of compound **3f**.

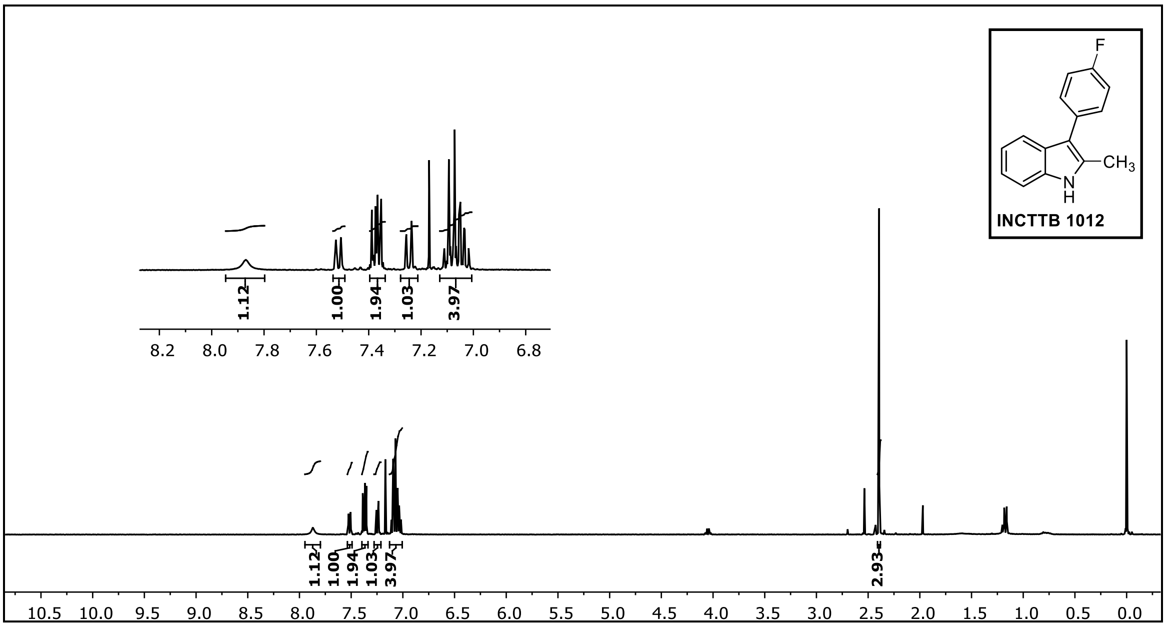

Figure S13 – ^{1}H NMR spectrum of compound **3g**.

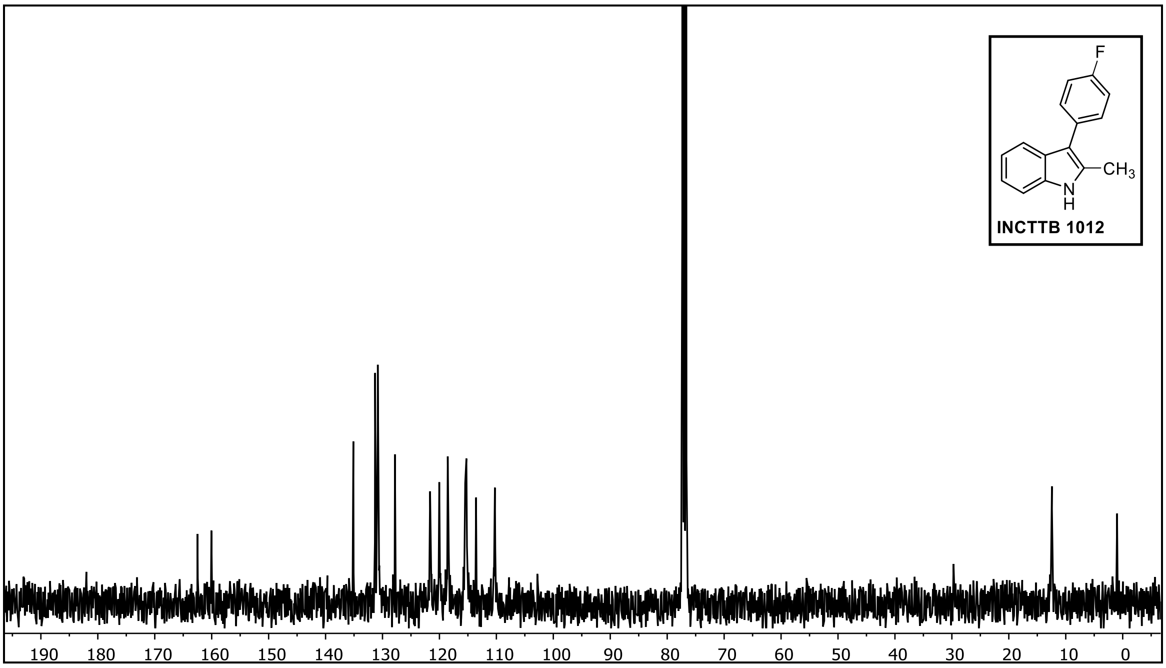

Figure S8 – ^{13}C NMR spectrum of compound **3g**.

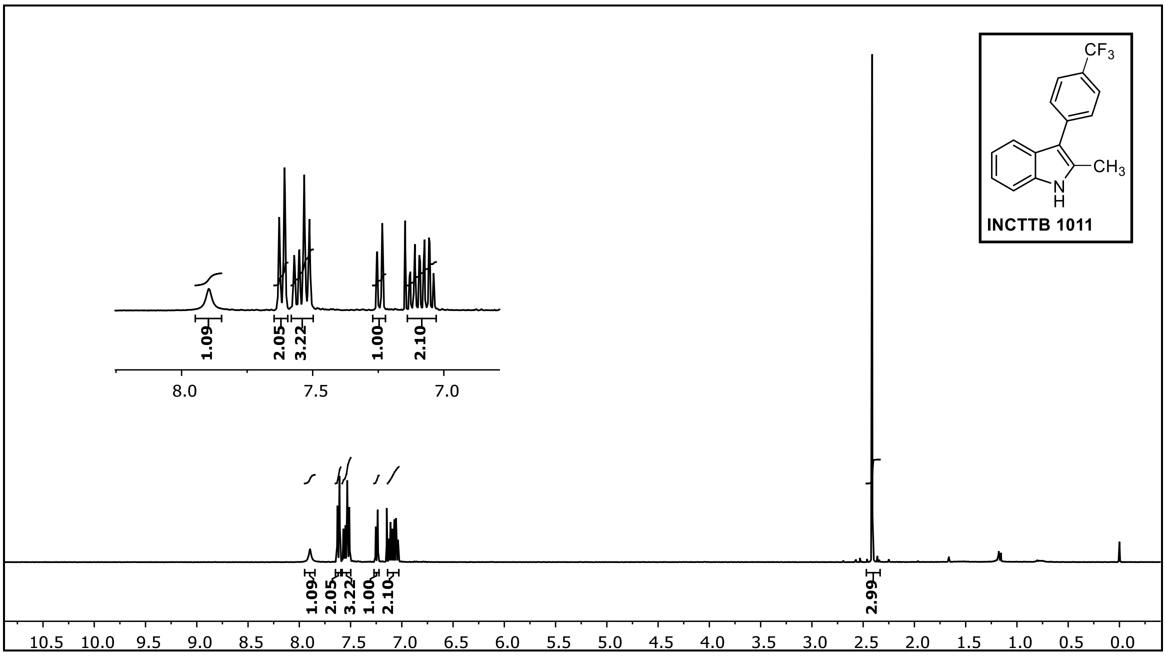

Figure S9 – ^{1}H NMR spectrum of compound **3h**.

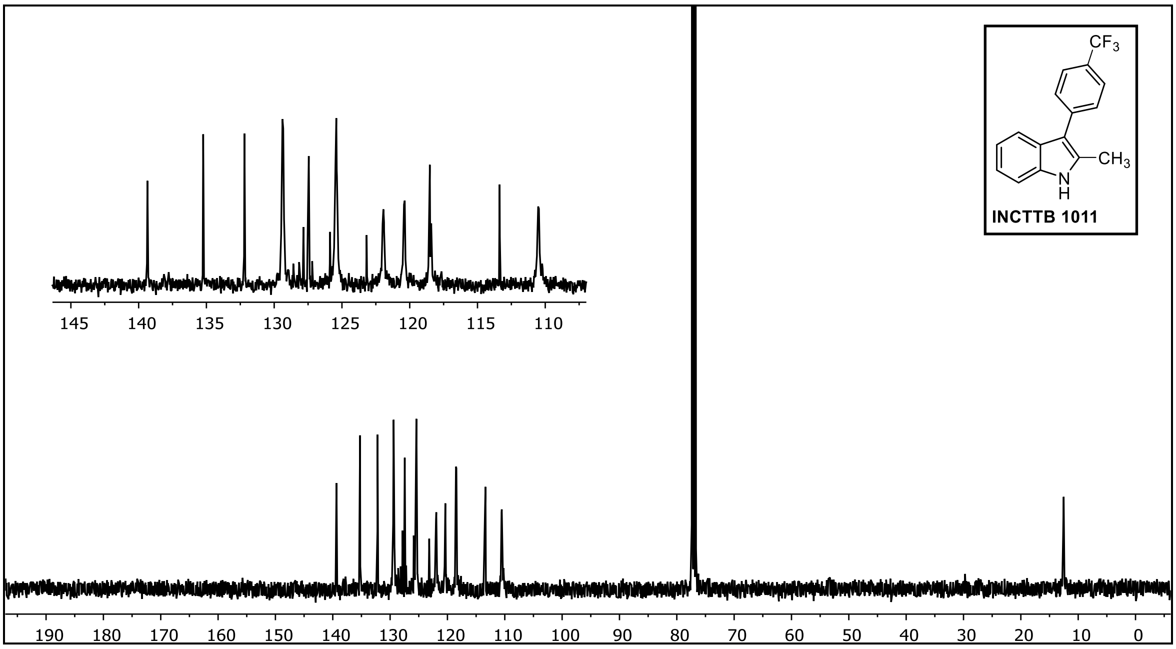

Figure S16 – ^{13}C NMR spectrum of compound **3h**.

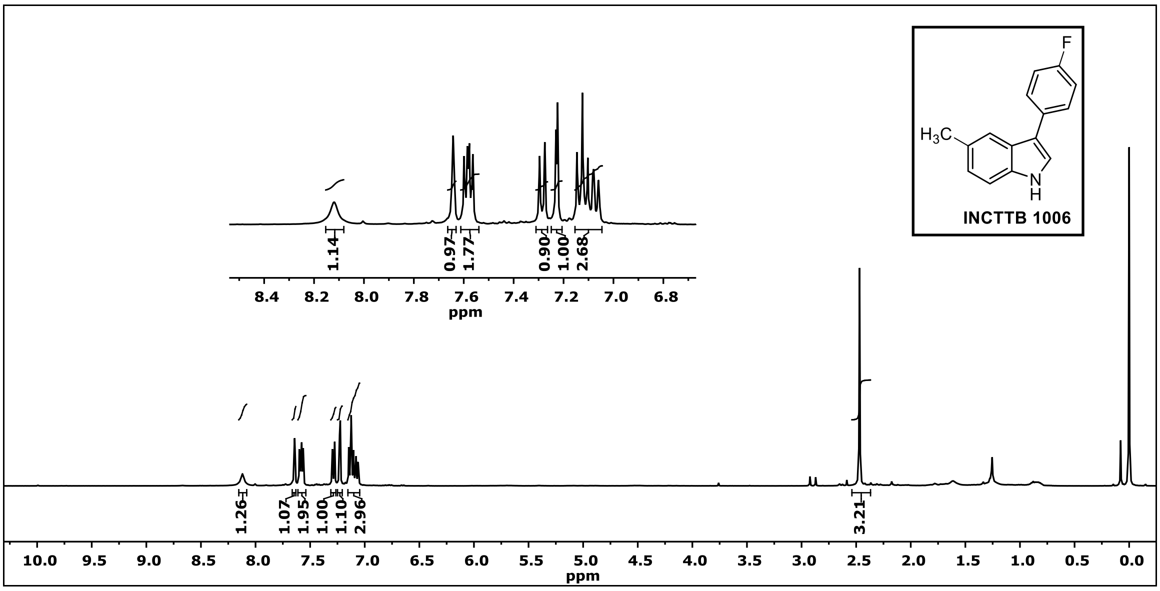

Figure S17 – ^{1}H NMR spectrum of compound **3i**.

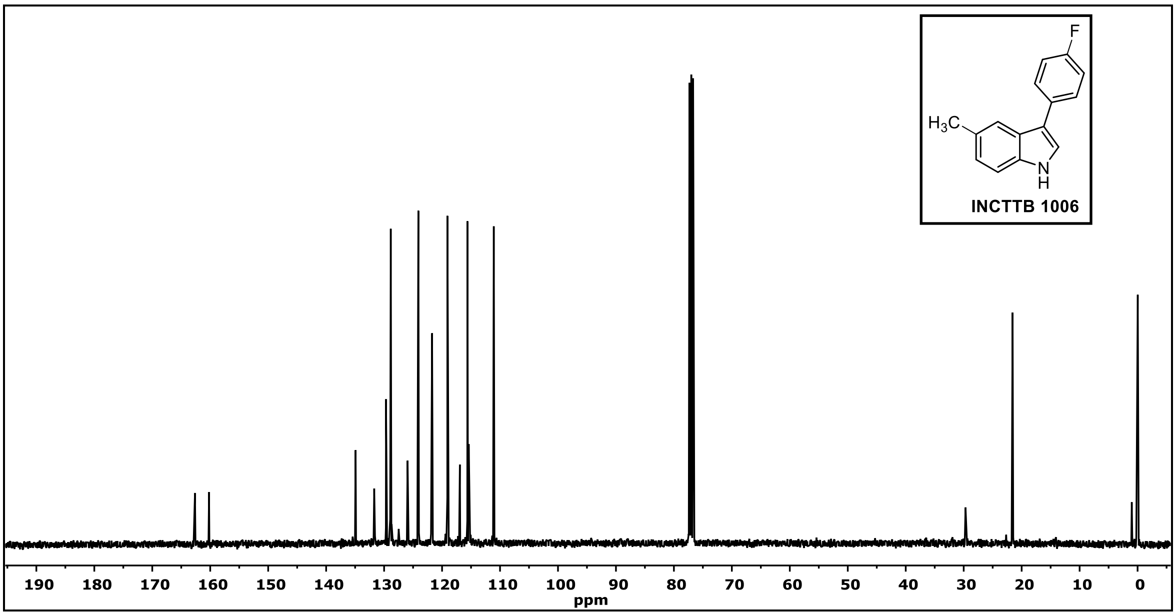

Figure S18 – ^{13}C NMR spectrum of compound **3i**.

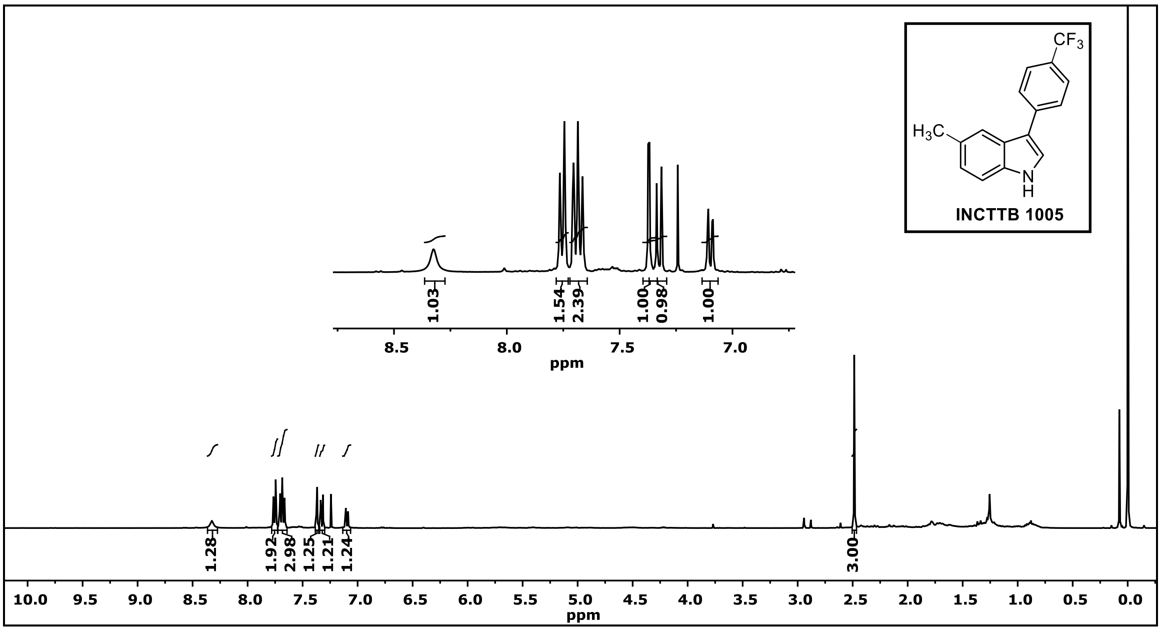

Figure S10 – ^{1}H NMR spectrum of compound **3j**.

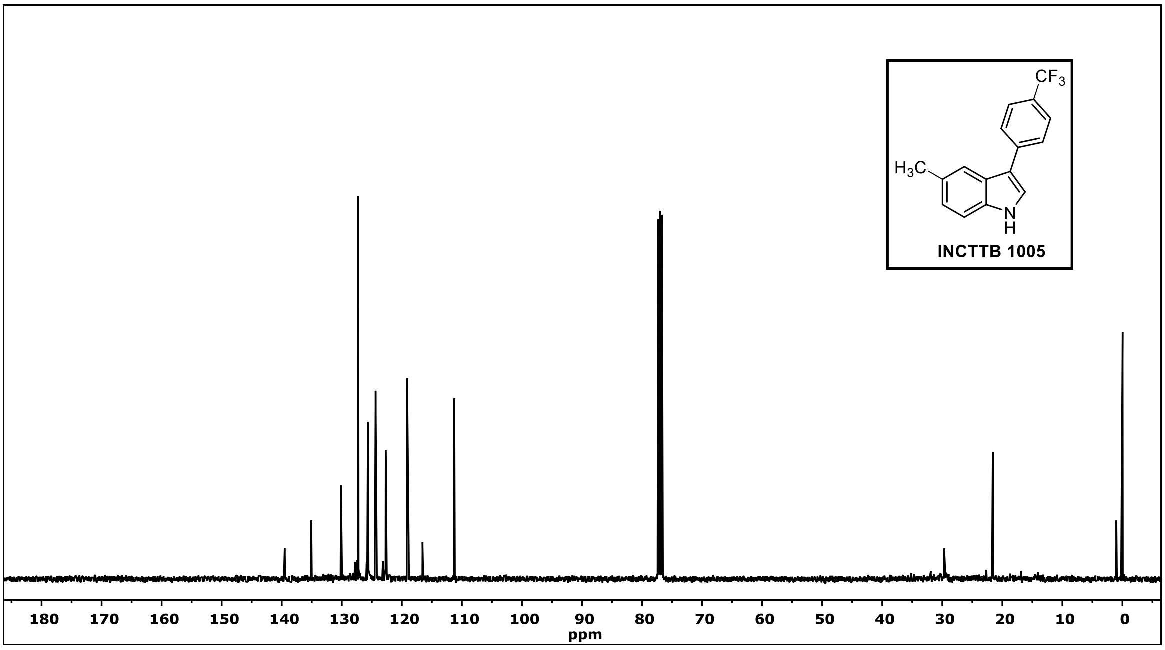

Figure S20 – ^{13}C NMR spectrum of compound **3j**.

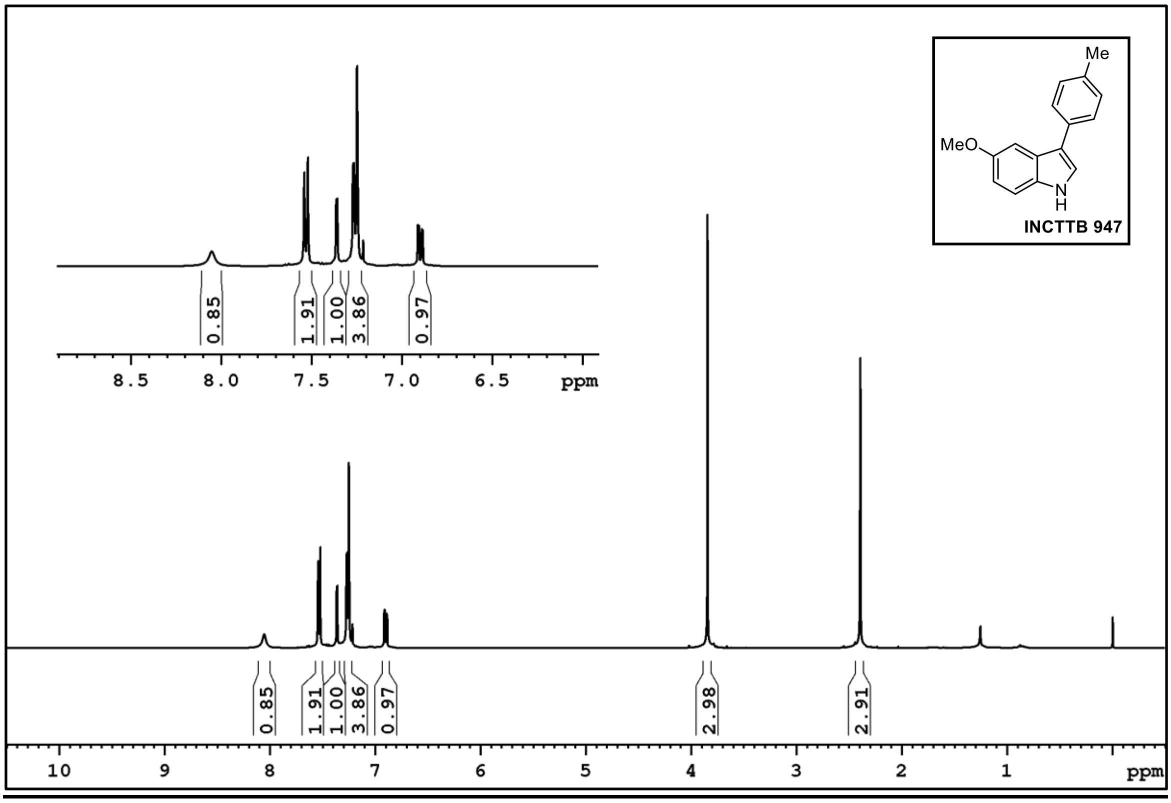

Figure S11 – ^{1}H NMR spectrum of compound **3k**.

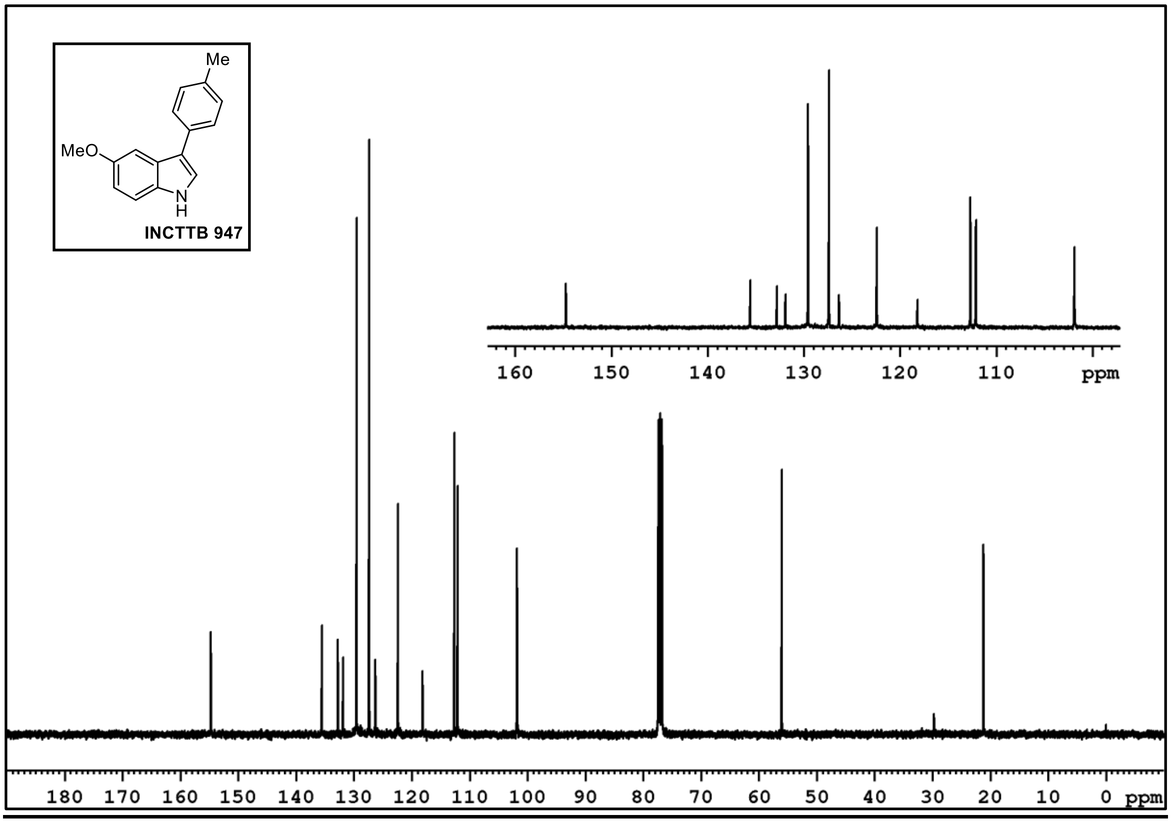

Figure S22 – ^{13}C NMR spectrum of compound **3k**.

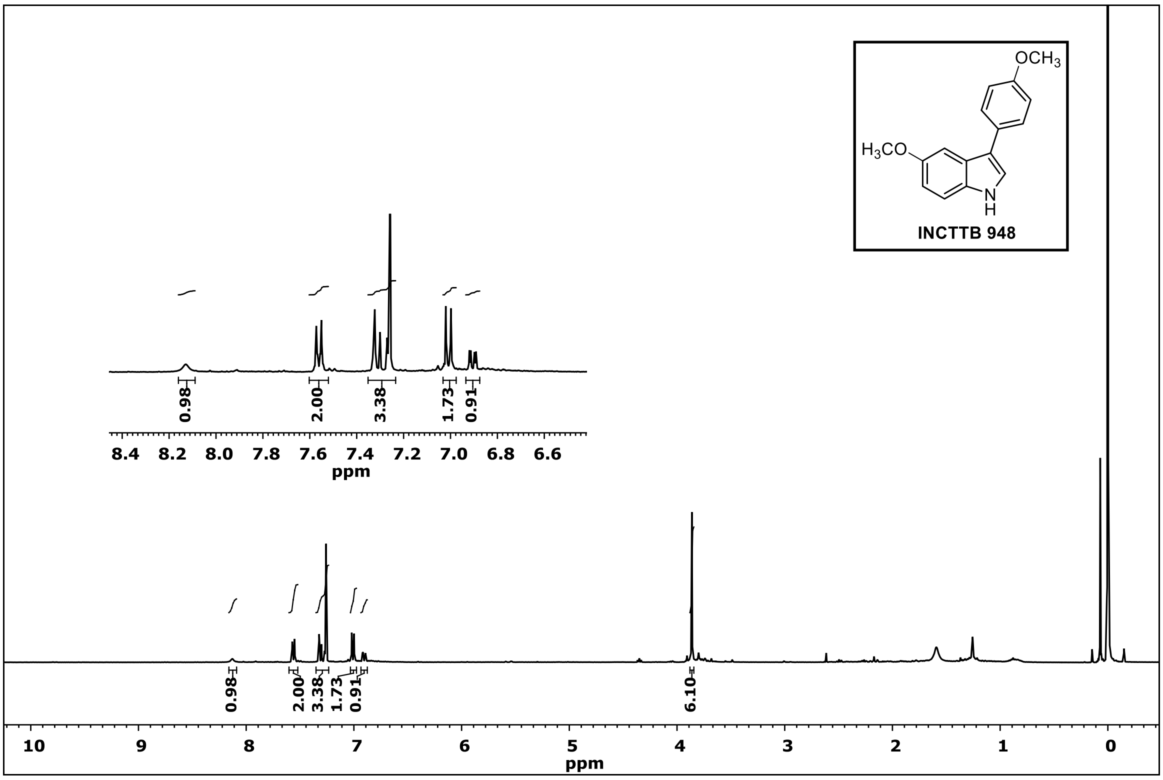

Figure S12 – ^{1}H NMR spectrum of compound **3l**.

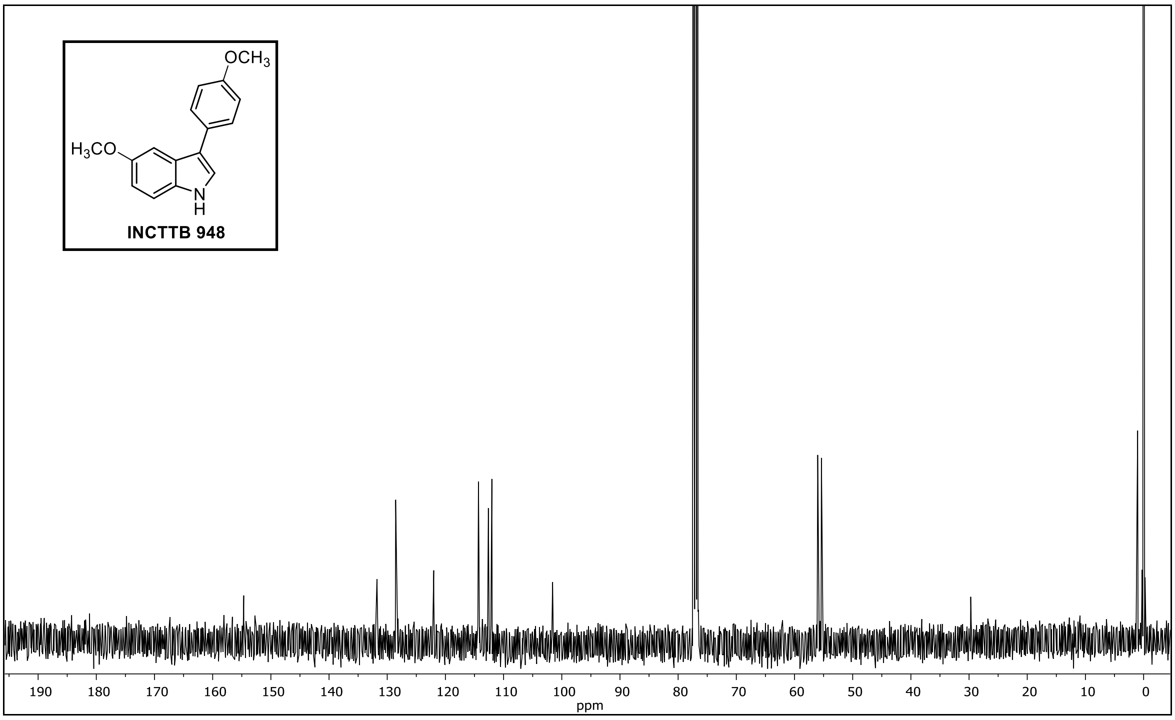

Figure S13 – ^{13}C NMR spectrum of compound **3l**.

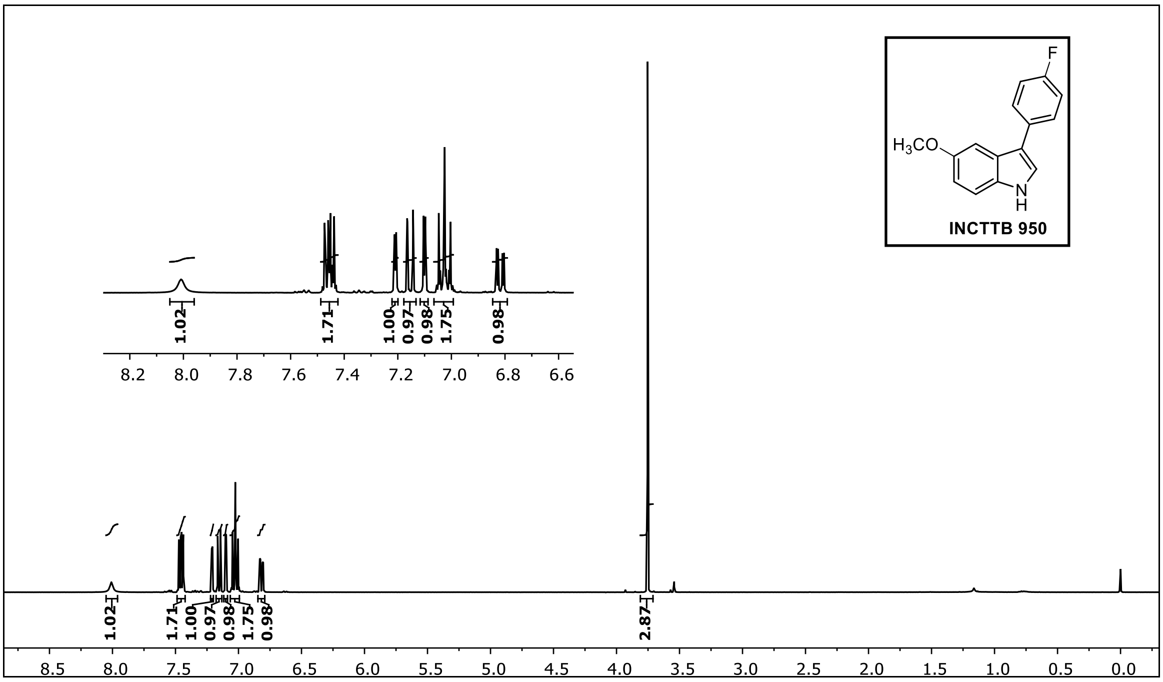

Figure S25 – ^{1}H NMR spectrum of compound **3m**.

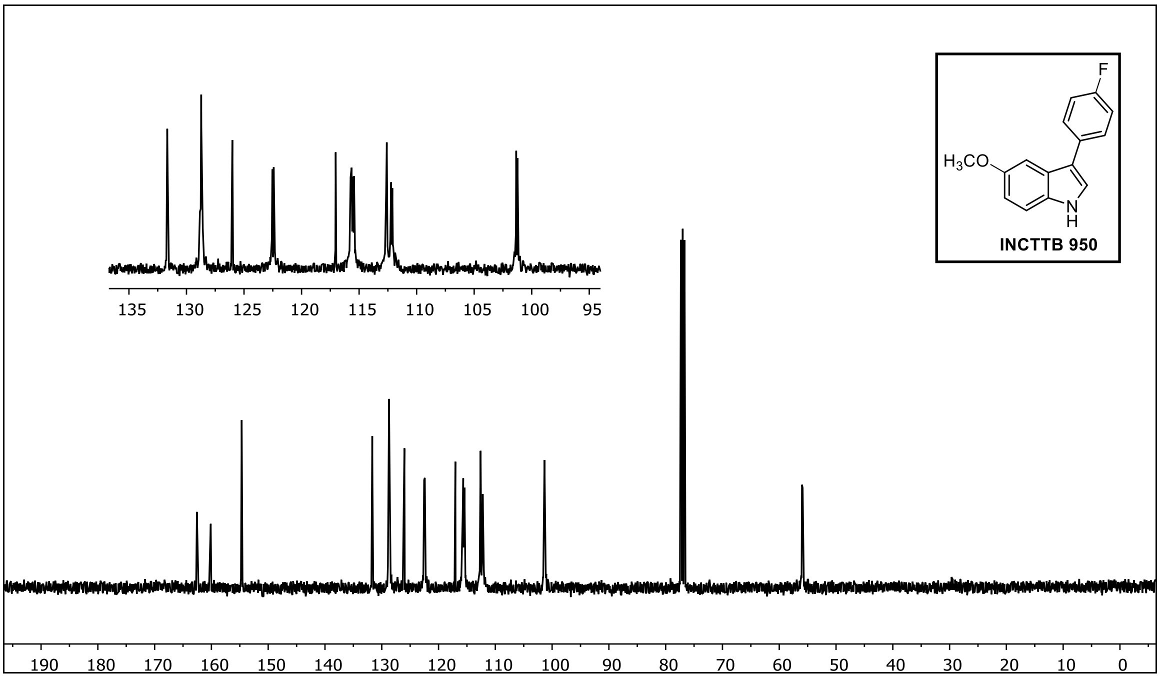

Figure S26 – ^{13}C NMR spectrum of compound **3m**.

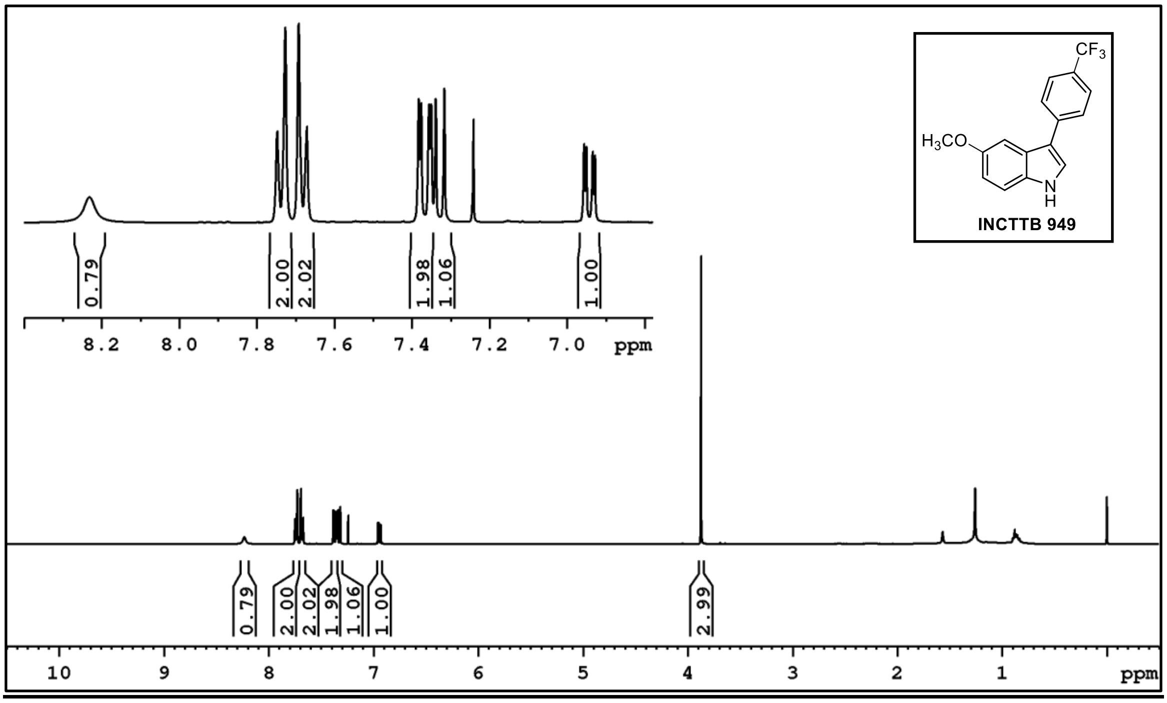

Figure S27 – ^{1}H NMR spectrum of compound **3n**.

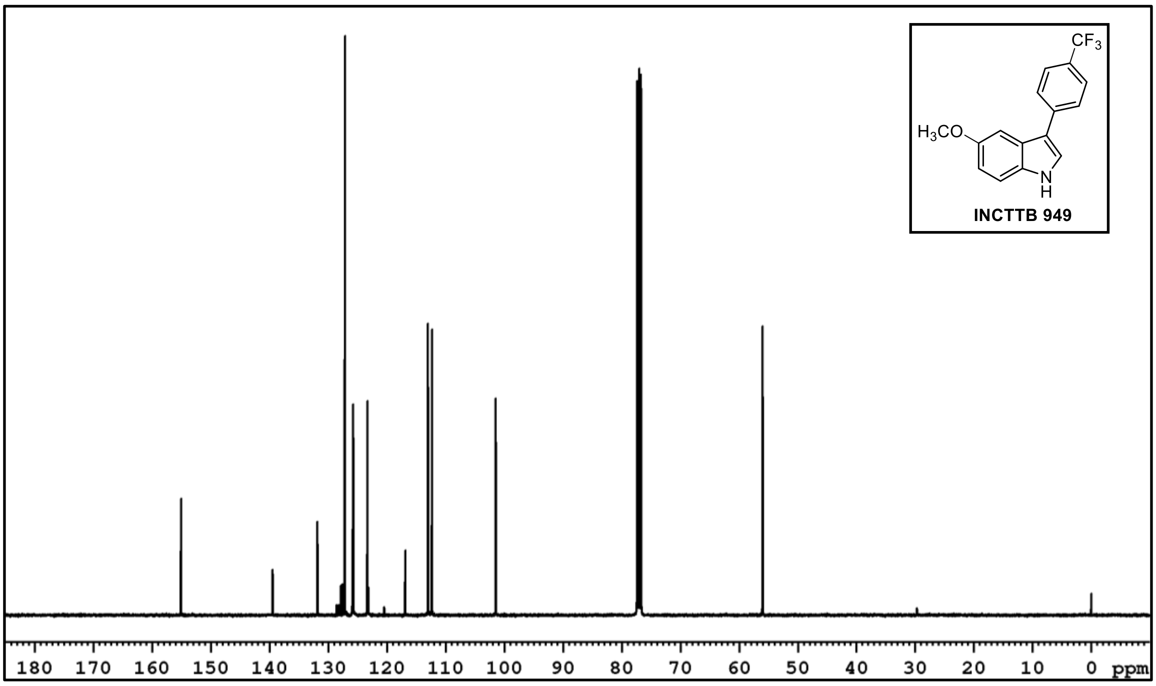

Figure S28 – ^{13}C NMR spectrum of compound **3n**.

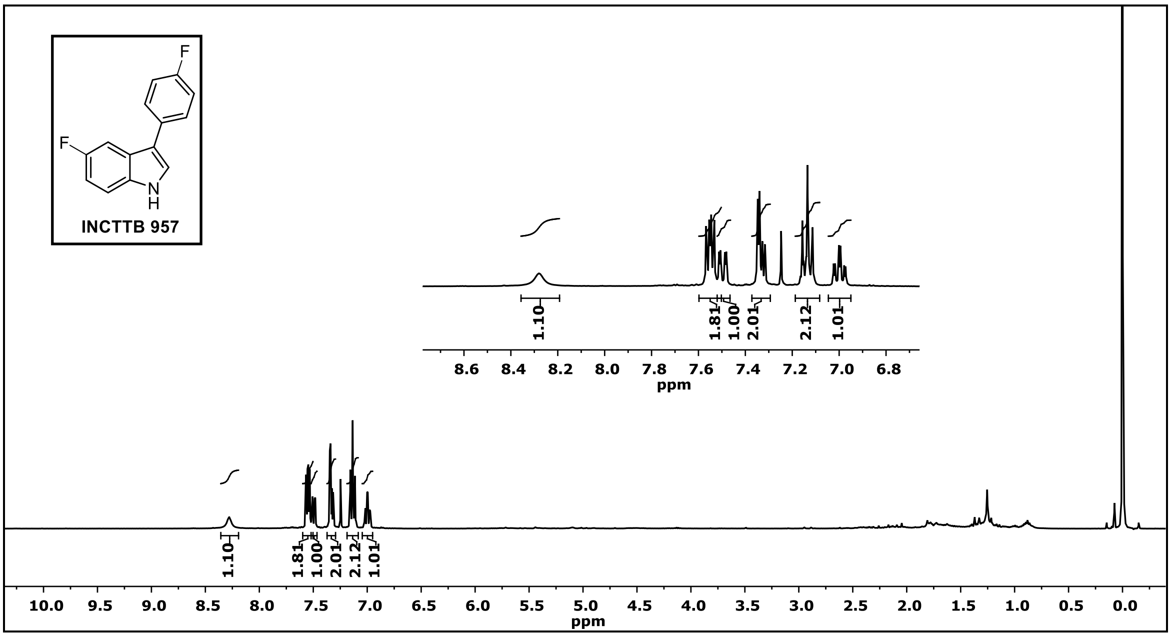

Figure S14 – ^{1}H NMR spectrum of compound **3o**.

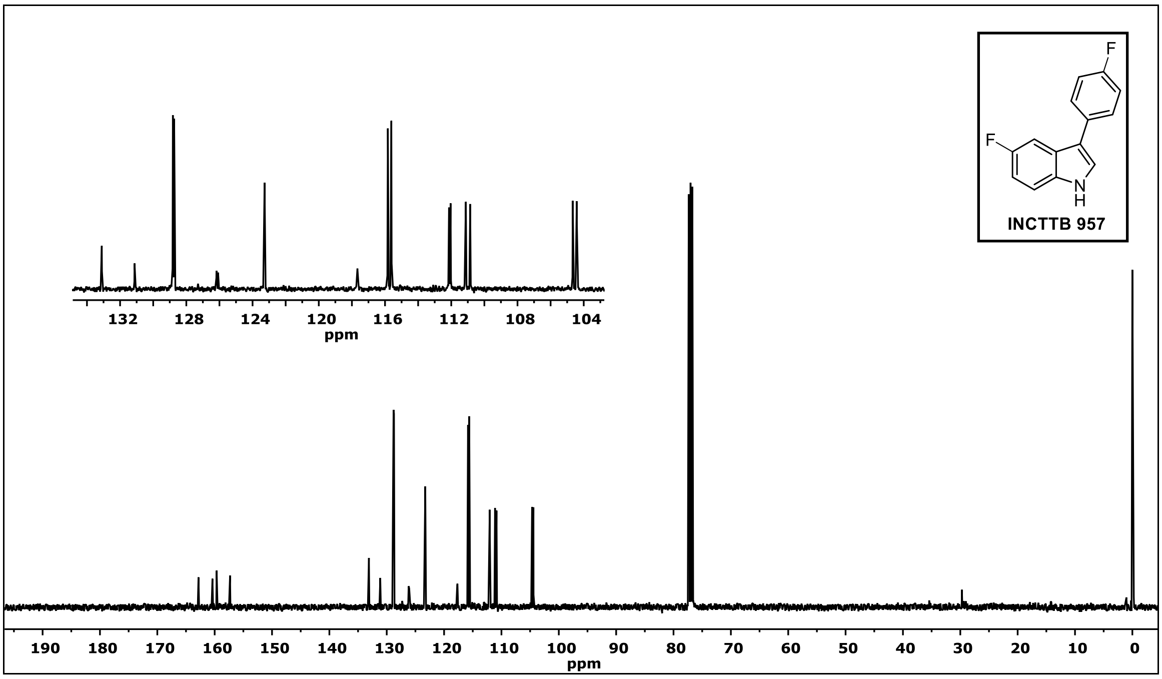

Figure S15 – ^{13}C NMR spectrum of compound **3o**.

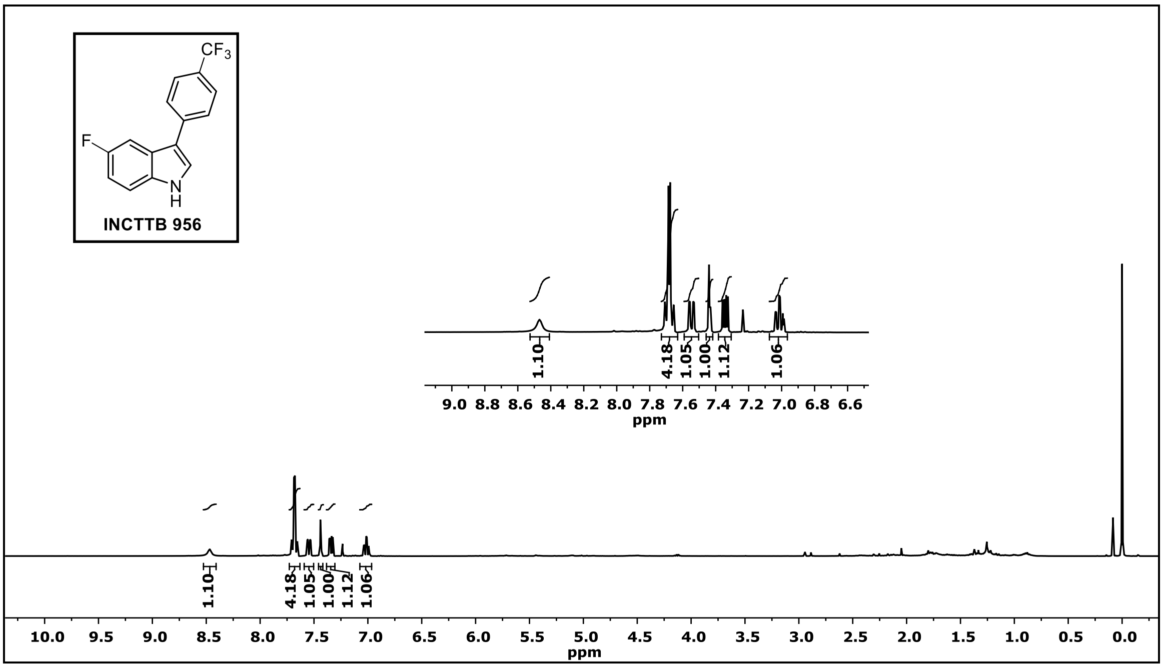

Figure S31 – ^{1}H NMR spectrum of compound **3p**.

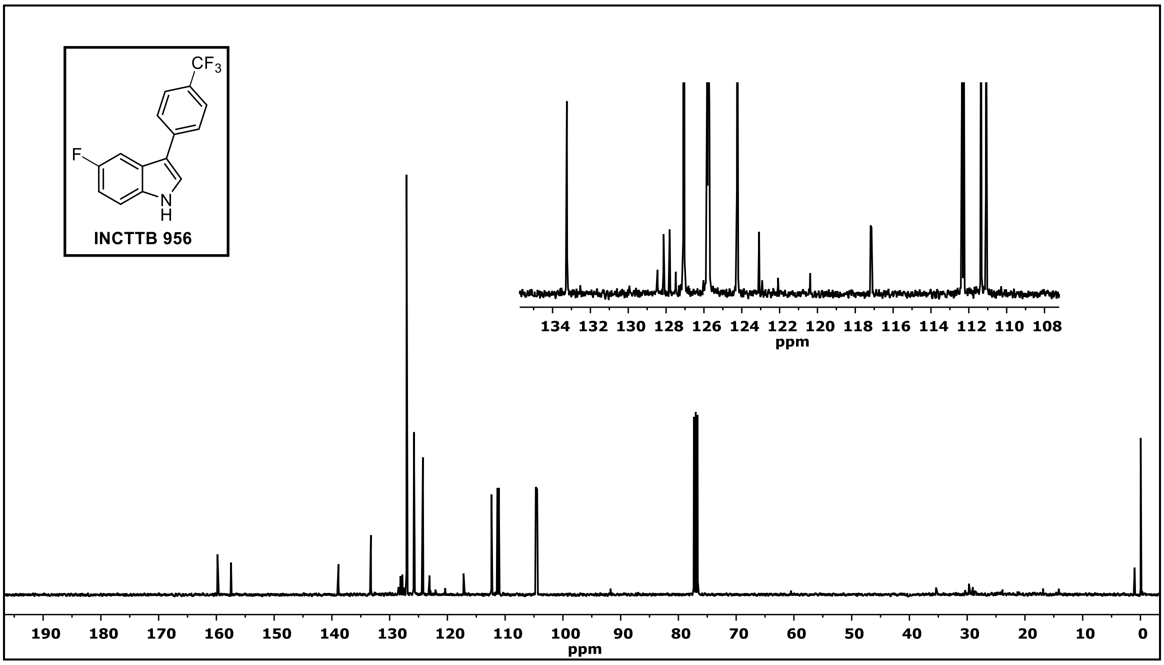

Figure S32 – ^{13}C NMR spectrum of compound **3p**.

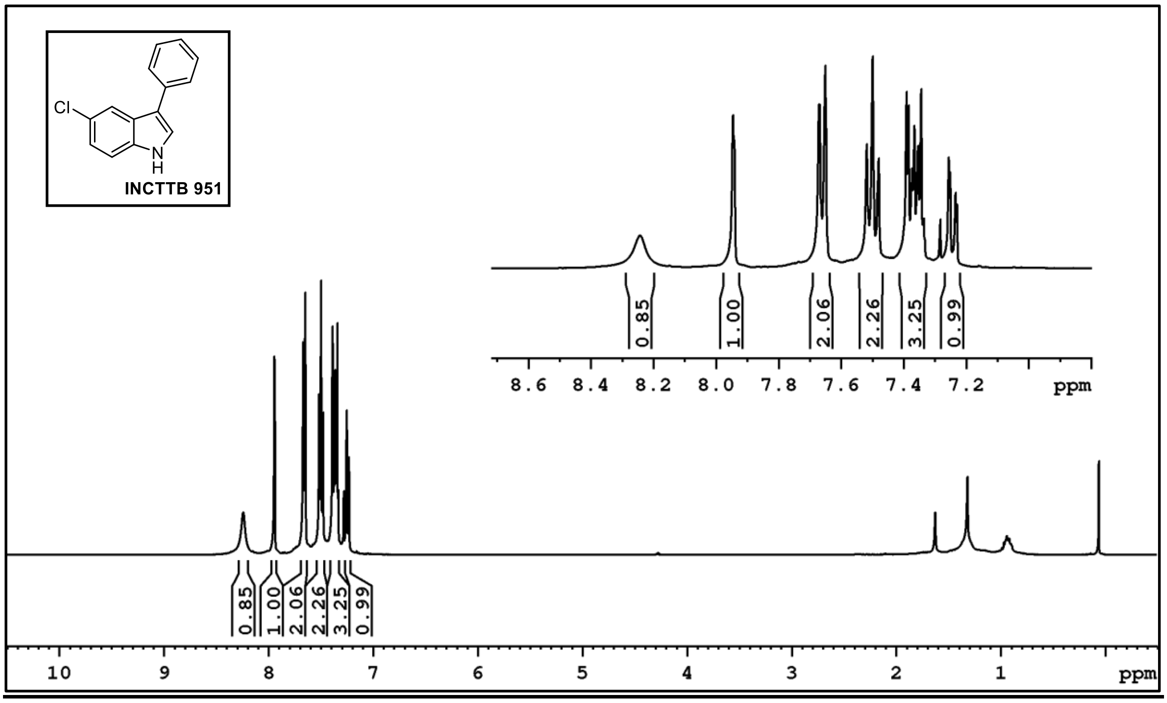

Figure S33 – ^{1}H NMR spectrum of compound **3q**.

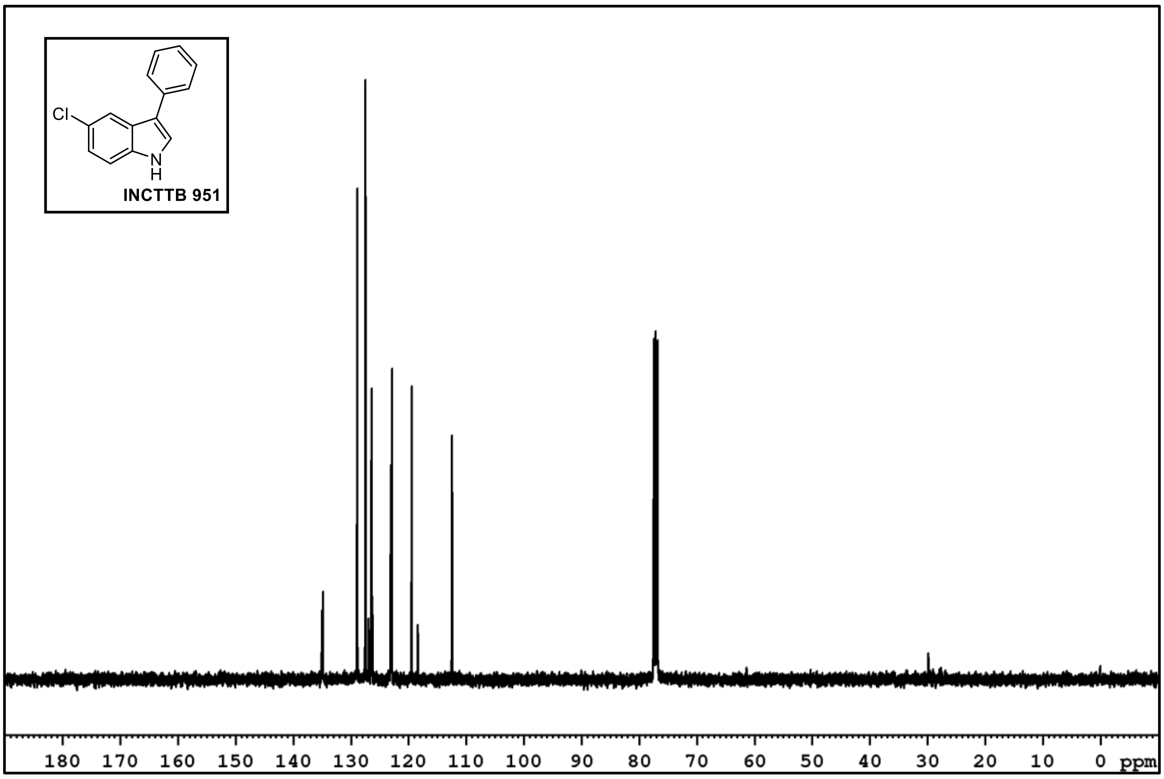

Figure S34 – ^{13}C NMR spectrum of compound **3q**.

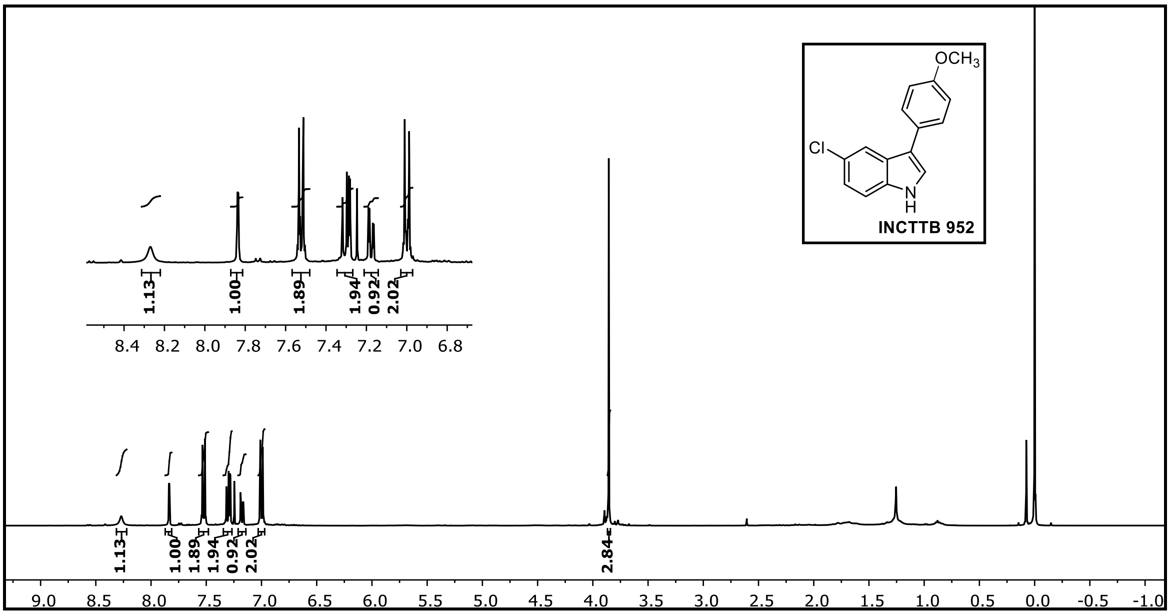

Figure S35 – ^{1}H NMR spectrum of compound **3r**.

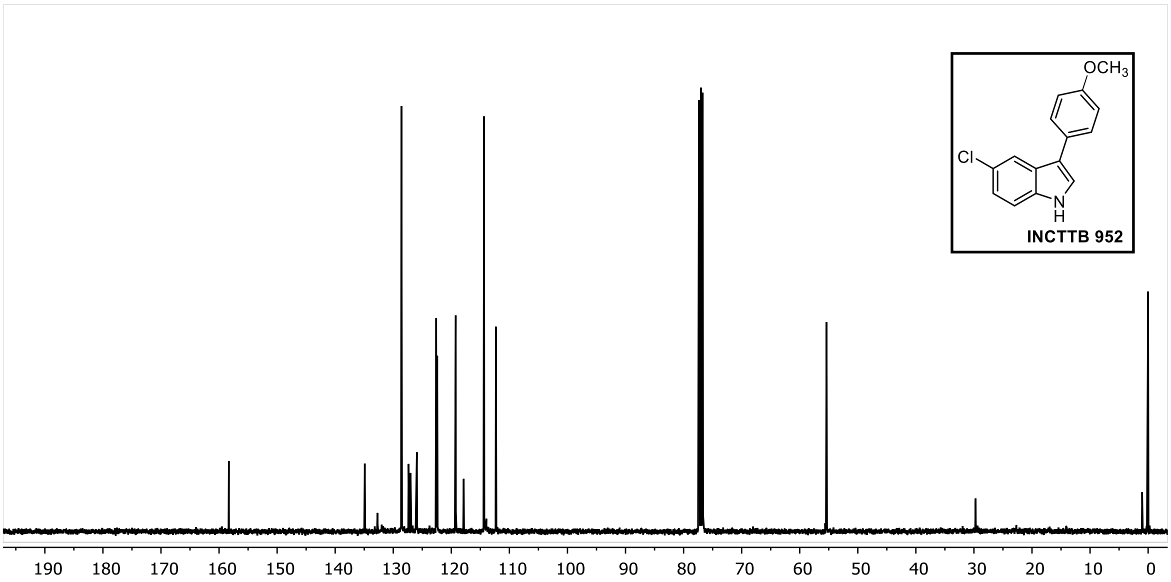

Figure S36 – ^{13}C NMR spectrum of compound **3r**.

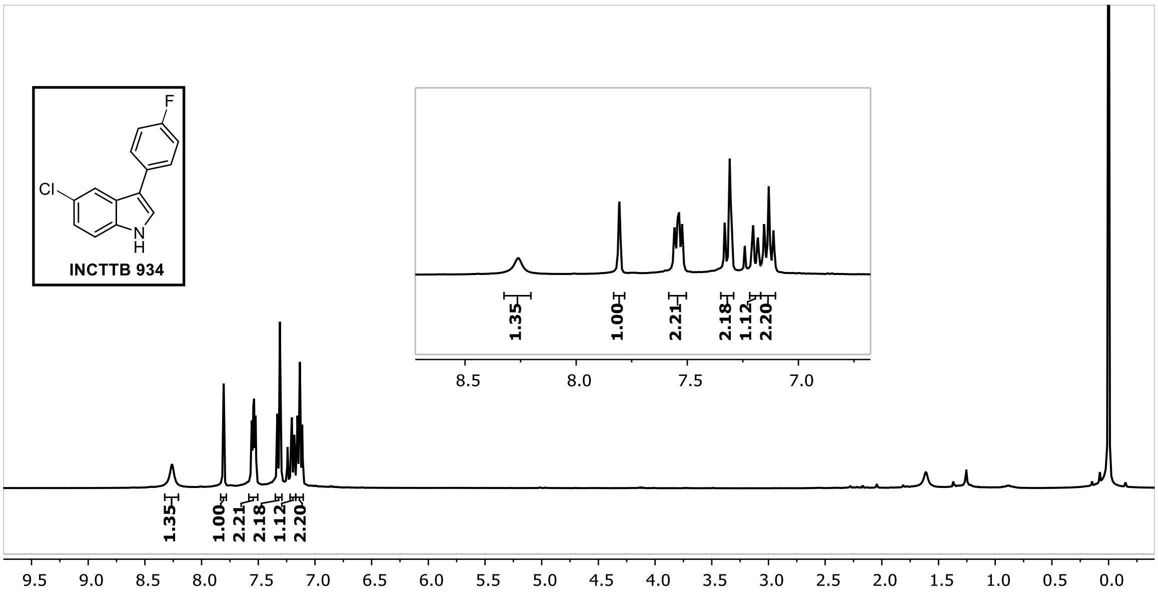

Figure S37 – ^{1}H NMR spectrum of compound **3s**.

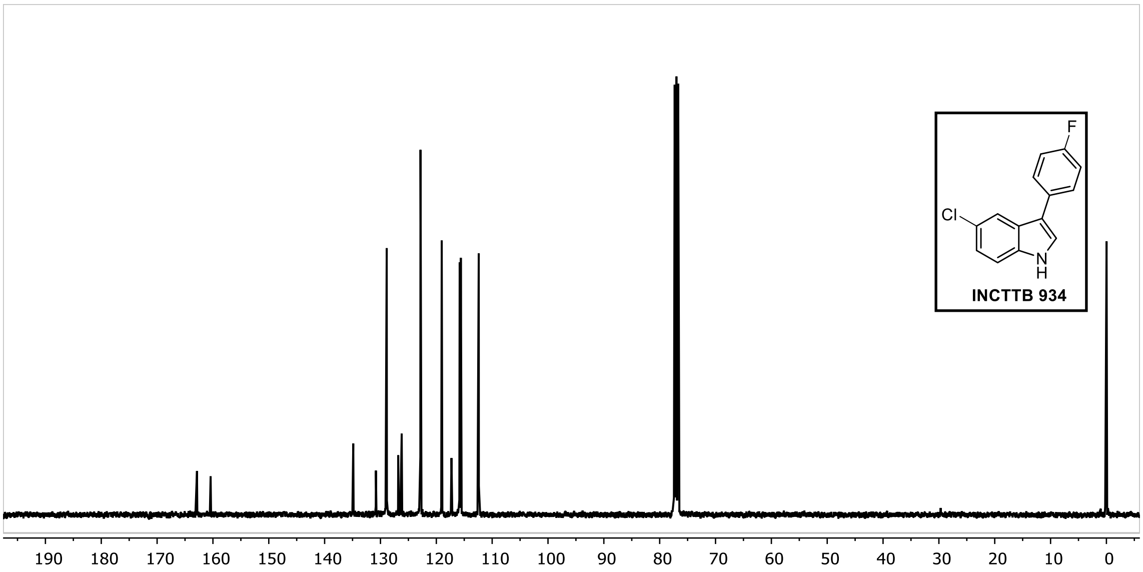

Figure S38 – ^{13}C NMR spectrum of compound **3s**.

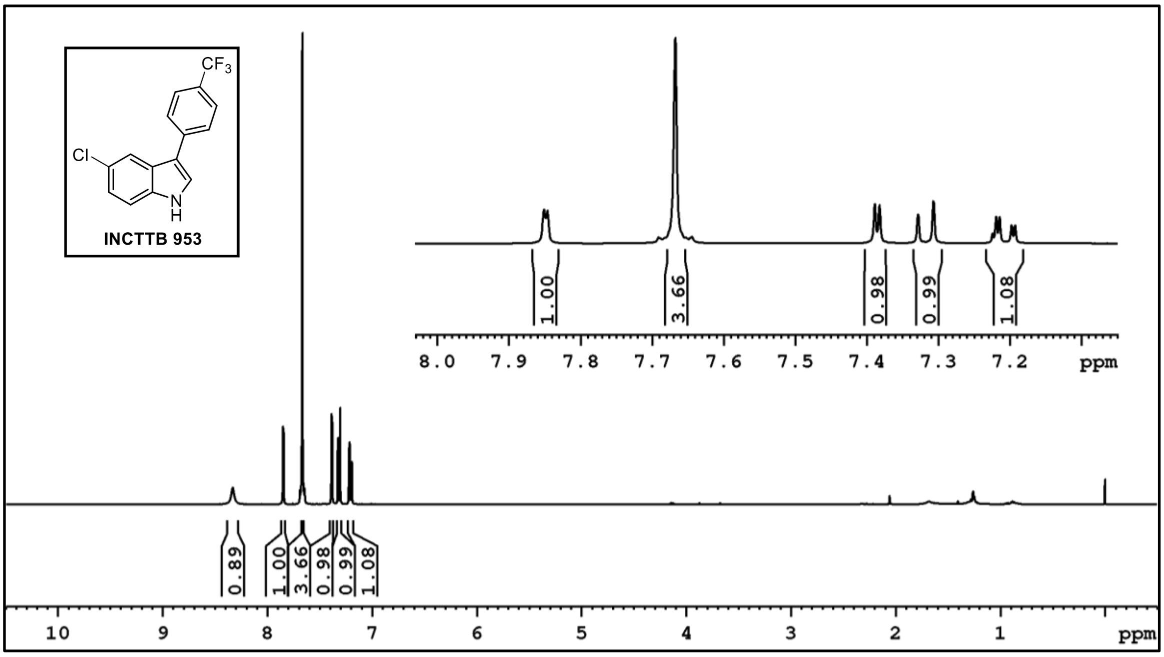

Figure S39 – ^{1}H NMR spectrum of compound **3t**.

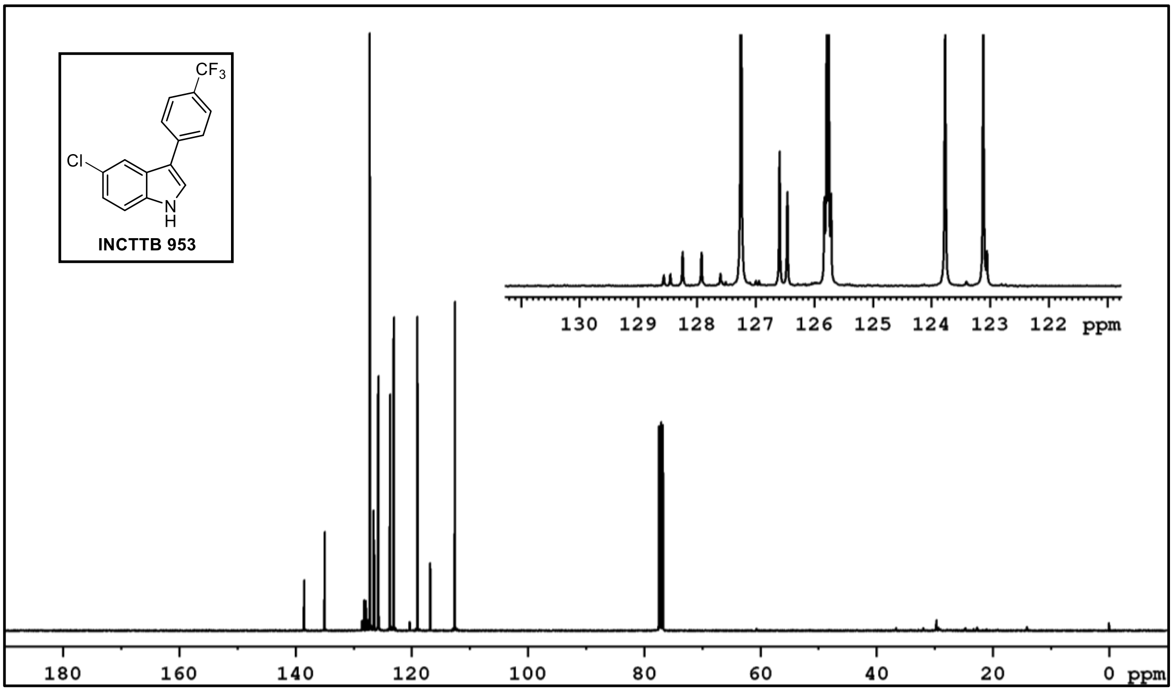

Figure S40 – ^{13}C NMR spectrum of compound **3t**.
